# Diversification of *FT-*like genes in the PEBP family contributes to the variation of flowering traits in Sapindaceae species

**DOI:** 10.1186/s43897-024-00104-4

**Published:** 2024-07-16

**Authors:** Xing Huang, Hongsen Liu, Fengqi Wu, Wanchun Wei, Zaohai Zeng, Jing Xu, Chengjie Chen, Yanwei Hao, Rui Xia, Yuanlong Liu

**Affiliations:** 1 State Key Laboratory for Conservation and Utilization of Subtropical Agro-Bioresources, South China Agricultural University, Guangdong Guangzhou, 510642 China; 2 South China Agricultural University, Guangdong Laboratory for Lingnan Modern Agriculture, South China Agricultural University, Guangdong Guangzhou, 510642 China; 3 South China Agricultural University, Key Laboratory of Biology and Germplasm Enhancement of Horticultural Crops in South China, Ministry of Agriculture and Rural Affair, South China Agricultural University, Guangdong Guangzhou, 510642 China; 4 College of Plant Science and Technology, Huazhong Agricultural University, Hubei Wuhan, 430070 China

**Keywords:** Sapindaceae, Lychee (*Litchi chinensis*), Flowering, FT1-like, *Cis*-regulatory element

## Abstract

Many species of Sapindaceae, such as lychee, longan, and rambutan, provide nutritious and delicious fruit. Understanding the molecular genetic mechanisms that underlie the regulation of flowering is essential for securing flower and fruit productivity. Most endogenous and exogenous flowering cues are integrated into the florigen encoded by *FLOWERING LOCUS T*. However, the regulatory mechanisms of flowering remain poorly understood in Sapindaceae. Here, we identified 60 phosphatidylethanolamine-binding protein-coding genes from six Sapindaceae plants. Gene duplication events led to the emergence of two or more paralogs of the *FT* gene that have evolved antagonistic functions in Sapindaceae. Among them, the *FT1*-like genes are functionally conserved and promote flowering, while the *FT2*-like genes likely serve as repressors that delay flowering. Importantly, we show here that the natural variation at nucleotide position − 1437 of the lychee *FT1* promoter determined the binding affinity of the SVP protein (LcSVP9), which was a negative regulator of flowering, resulting in the differential expression of *LcFT1*, which in turn affected flowering time in lychee. This finding provides a potential molecular marker for breeding lychee. Taken together, our results reveal some crucial aspects of *FT* gene family genetics that underlie the regulation of flowering in Sapindaceae.

**Supplementary Information:**

The online version contains supplementary material available at 10.1186/s43897-024-00104-4.

## Core

The natural variation at nucleotide position − 1437 of the lychee *FT1* promoter determined the binding affinity of the SVP protein (LcSVP9), a negative regulator of flowering, resulting in the differential expression of *LcFT1*, which in turn affected flowering time in lychee. This finding provides a potential molecular marker for lychee breeding.

## Gene and accession numbers

Sequence data of Sapindaceae plants from this article can be found in the SapBase database (http://www.sapindaceae.com/Download.html)(Li et al. 2024). The remaining sequences were obtained from the Phytozome, GenBank, and Arabidopsis Information Resource (TAIR) databases. The accession numbers can be found in Table S2 and Table S6.

## Introduction

Lychee (*Litchi chinensis*), longan (*Dimocarpus longan*), and rambutan (*Nephelium lappaceum*) are commercially important fruit trees in the Sapindaceae family, which are extensively cultivated in tropical and subtropical areas worldwide (Menzel et al. 1995; Shahrajabian et al. 2020). These species are closely related and possess valuable fruits with an edible aril (Zee et al. 1998). Although they are closely related, their flowering traits, which are directly linked to production, are not entirely identical. Lychee and longan are day-neutral plants that require prolonged exposure to low temperatures to consistently flower annually (Subhadrabandhu et al. 2000; Chen et al. 2003). In contrast, rambutan flowering is triggered by water scarcity, which occurs biannually during March to May and July to August in reaction to two short periods of arid conditions followed by intermittent rainfall (Shaari et al. 1983; Tindall, 1994  ). Longan exhibits a unique floral induction mechanism in response to potassium chlorate (KClO_3_), enabling off-season and year-round fruit production, which is unparalleled in other fruit crops (Matsumoto et al. 2007). Understanding the molecular genetic processes that control flowering would help the Sapindaceae fruit industry.

Plants undergo significant physiological changes during the transition from vegetative phase to reproductive stage, which is triggered by various internal and external signals that ultimately lead to flowering. The regulatory mechanisms of flowering in model plants have been elucidated by identifying at least five genetically defined pathways (Srikanth et al. 2011). The components of these pathways can vary across species, but most internal and external signals are integrated into a few central hubs that are conserved, the most well-known of which is the florigen encoded by *Flower Locus T* (*FT*), which belongs to the phosphatidylethanolamine-binding protein (PEBP) family (Kardailsky et al. 1999; Liu et al. 2016). In plants, FT serves as a small, mobile signaling molecule that is synthesized in the leaves and moves to the shoot apex, enabling the transition to reproductive development and flowering (Corbesier et al. 2007; Mathieu et al. 2007). Despite the considerable conservation observed in flowering regulators among plant species, recent research has revealed cases where FT homologs have evolved to inhibit flowering (Blackman et al. 2010; Pin et al. 2010; Harig et al. 2012; Lee et al. 2013; Nan et al. 2014; Zhai et al. 2014). The presence of four FT homologs has been identified in the Sunflower (*Helianthus annuus*), with one of them exhibiting a novel repressor function attributed to a mutation (Blackman et al. 2010). Similarly, sugar beet contains the BvFT1 and BvFT2 homologs of FT with antagonistic functions (Pin et al. 2010). External loop formation of BvFT caused by the divergence of the amino acids within segment B may elucidate the opposite functions (Pin et al. 2010). The Y134 and W138 residues within segment B act to change the FT function (Pin et al. 2010). The MADS-box genes, *SHORT VEGETATIVE PHASE* (*SVP*) and *SUPPRESSOR OF OVEREXPRESSION OF CONSTANS 1* (*SOC1*), encode essential transcription factors that play a crucial role in regulating the integration of flowering signals (Ng and Yanofsky 2011; Moon et al. 2003; Kou et al. 2022). SVP directly binds to the CArG motifs of *FT* and its homolog, repressing their expression in leaves (Jang et al. 2009). In contrast, SOC1 activates *FT* transcription in the leaf vasculature of soybean by directly binding to the CArG motif within its promoter; thus, ensuring the induction of flowering (Kou et al. 2022). SOC1 and SVP bind to CArG boxes, but their binding preferences vary. SOC1 predominantly binds to the SRF-type CArG box consensus sequence, while SVP binds to the intermediate type (Tao et al. 2012).

In contrast to mutations that impair gene function in protein-coding regions, variations occurring in either *cis*-regulatory elements (CREs) or regulatory regions result in alterations of transcriptional level that impact the phenotype. Variations in the *MdIPT5b* gene promoter region of apples results in a marked increase in cellular cytokinin levels (Feng et al. 2021). This can be attributed to the deletion of a 42-bp sequence within the promoter region, which impairs the *cis*-element ProRE (an ACTCAT motif), leading to stable gene expression under salt stress conditions. A variation (single nucleotide polymorphism (SNP)13 T/C) in the regulatory region of PbCPK28, a SNP-13, leads to variations in the fructose content of pear (Li et al. 2023). Similar regulatory mechanisms have been confirmed in the regulation of cold tolerance in tomatoes (Zhu et al. 2023), grain width and weight in rice (Ruan et al. 2020), and low-temperature tolerance in maize (Jiang et al. 2022). The variations in the promoter region have the potential to drive evolution, generating genetic variability that can be harnessed for domestication and breeding advances (Swinnen et al. 2016).

Despite the mechanisms of floral regulation have been thoroughly investigated in model species, such as arabidopsis (*Arabidopsis thaliana*) (Jang et al. 2009), tomato (*Solanum lycopersicum*) (Huang et al. 2021; Chong et al. 2022), and rice (*Oryza sativa*) (Pan et al. 2022; Tang et al. 2023), they remain poorly understood in the Sapindaceae family. Therefore, it is essential to comprehend the regulatory mechanisms that govern flowering in Sapindaceae fruit trees. Understanding the regulation of flowering time in Sapindaceae is crucial for efficient breeding and improving commercial production. Thus, in our study, we used genomic data from six Sapindaceae species and massive RNA-seq data to carry out a genome-wide characterization of the FT family in Sapindaceae and explore the variations in the coding and promoter regions of the *FT* genes, which contribute to the variation of flowering traits in Sapindaceae species.

## Results

### Gene member variations of the PEBP gene family in *Sapindaceae*

**Fig. 1:**
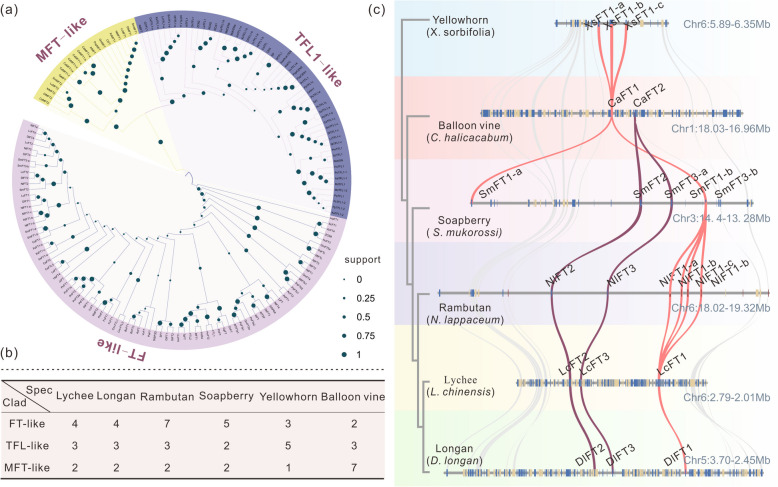
The variation in gene numbers within the PEBP gene family across six Sapindaceae plants. **a** Phylogenetic analysis of the PEBP protein family in 52 flowering plant species. The construction of the tree was performed utilizing the Neighbor Joining in MEGA X (Kumar et al. 2018). **b** The PEBP gene copy numbers in each species were mapped in this table. “Clad” and “spec” are short for “caldes” and “species” respectively. **c** Macrosynteny across lychee, longan, rambutan, soapberry, yellowhorn and balloon vine for *FT*-like genes. Syntenic blocks are connected by lines. The gene pairs of *FT1*-like are linked with red curves, while those of *FT2*-like/*FT3*-like are connected by purple curves

To investigate the composition and evolutionary relationships in the PEBP proteins from Sapindaceae, we screened the genomes of six Sapindaceae species with available genomes, including lychee (*Litchi chinensis*), longan (*Dimocarpus longan*), rambutan (*Nephelium lappaceum*), soapberry (*Sapindus mulorossi*), yellowhorn (*Xanthoceras sorbifolium*), and balloon vine (*Cardiospermum halicacabum*). Sixty PEBP proteins with complete open reading frames were identified in Sapindaceae (Fig. 1a, b, Fig. S1 and Table S1). We performed a phylogenetic analysis with 96 functionally reported PEBP proteins from 46 other major angiosperm lineages (Wickland et al. 2015; Table S2), which indicated that the PEBP genes were separated into three primary clusters of MFT-like, TFL1-like, and FT-like clades (Fig. 1a). The number of PEBP genes in Sapindaceae varied, ranging from nine to twelve copies (Fig. 1b). Lychee and longan possessed four *FT* genes, but there were seven *FT*-like genes in rambutan, which is a close relative of lychee and longan (Fig. 1b), indicating that *FT* gene expansion occurs in rambutan. Fewer *FT*-like genes were detected in balloon vine than in the other five woody Sapindaceae species, however, the *MFT*-like genes were expanded (Fig. 1b).

To investigate the evolutionary relationships among the *FT*-like genes, a syntenic analysis was performed on the Sapindaceae family. The *FT1* genes maintained a good syntenic relationship across Sapindaceae, indicating that *FT1* is functionally conserved. The *FT2* genes lost their syntenic relationship in the basal Sapindaceae plant, yellowhorn, but they were conserved in the other Sapindaceae plants. The *FT1*, *FT2*, and *FT3* loci maintained good collinearity in lychee, longan, rambutan, and soapberry (Fig. 1c). In particular, the *FT1* gene was tandemly duplicated into four copies in rambutan, which led to the expansion of the *FT* genes (Fig. 1c). A similar tandem replicate event occurred in *FT1* loci of yellowhorn (Fig. 1c), and *MFT1* loci of balloon vine (Fig. S2). These results suggest that tandem duplication events contribute to PEBP gene expansion in Sapindaceae.

### Functional antagonism of *FT* homologs in floral induction

**Fig. 2:**
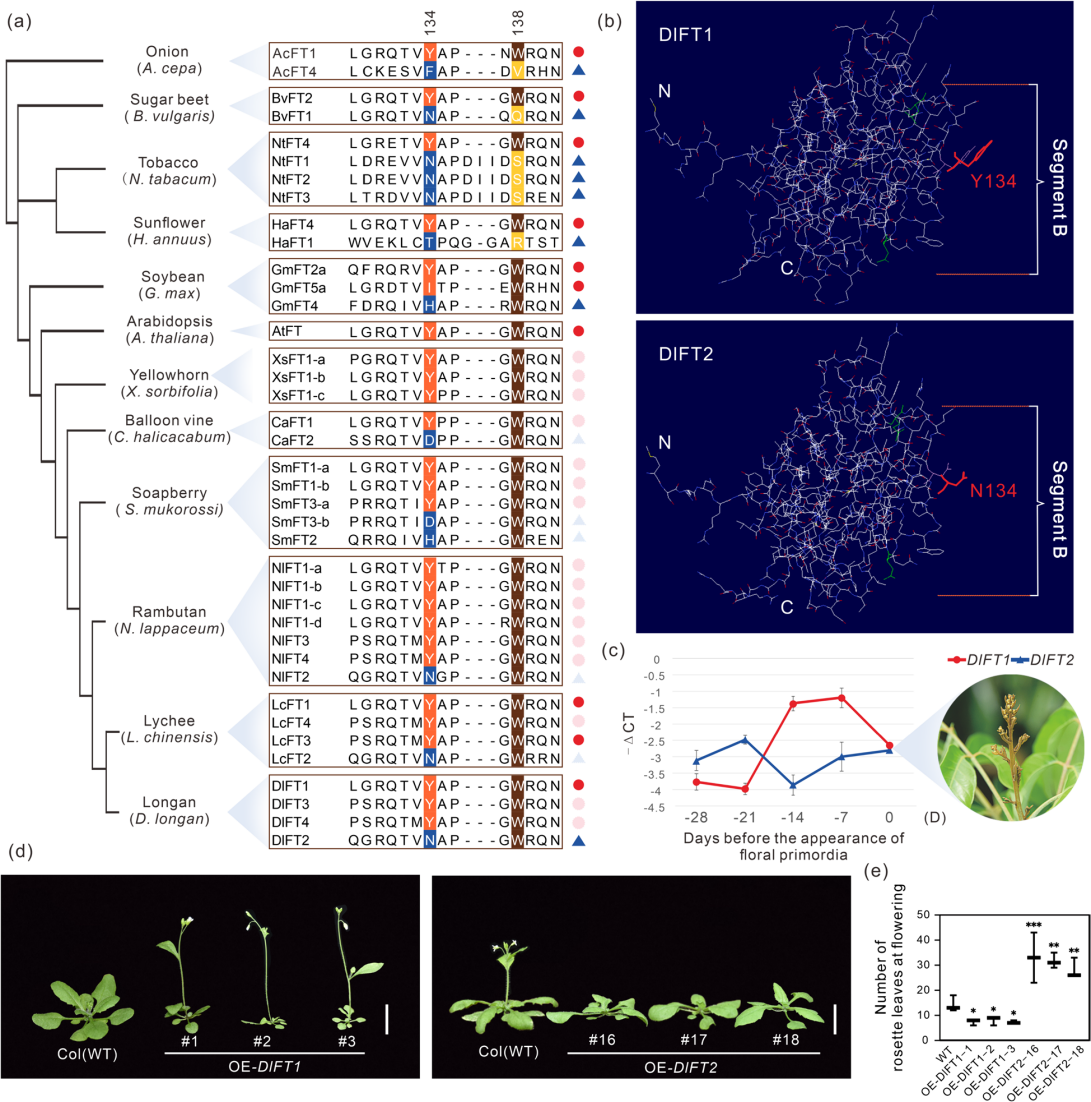
Investigating the crucial regions contributing to the antagonistic functions of FTs. **a** The alignment of the amino acid sequence in the fourth exon for the FT inducer and inhibitor from onion (Lee et al. 2013), sugar beet (Pin et al. 2010), tobacco (Harig et al. 2012), sunflower (Blackman et al. 2010), soybean (Nan et al. 2014; Zhai et al. 2014; Kong et al. 2010), arabidopsis (Kardailsky et al. 1999; Kobayashi et al. 1999), lychee (Ding et al. 2015), longan (Winterhagen et al. 2013), rambutan, soapberry, yellowhorn and balloon vine. Red dots indicate inducers that have been functionally validated, while blue triangles indicate repressors that have undergone functional validation. Pink dots represent speculated activators, while light blue triangles represent speculated inhibitors. **b** The prediction of protein folding for DlFT1 and DlFT2. The molecular structures were visualized using solid three-dimensional traces in a diverse color scheme for alignment. **c** The expression pattern of *FTs* during flower induction in longan leaves was analyzed using quantitative real-time polymerase chain reaction (qRT-PCR). The − delta CT (− ΔCT) calculation was employed to normalize expression levels. The data presented represent the mean ± standard deviation (SD) from three independent biological replicates, with *Actin-100* (Dil.11g013910.1) used as the internal control gene. **d** The phenotype of *Arabidopsis* with the overexpression of longan *FT1* and *FT2* genes. All plants were grown under long-day conditions with 16 h of light and 8 h of darkness at 23℃. The left image depicts the phenotype after 26 days of sowing, while the right image showcases the phenotype after 30 days of sowing. Col (WT) represents the wild-type *Arabidopsis* with a Col-0 background. OE represents the transgenic lines with *DlFT* overexpression. T_2_ generation lines with *DlFT1* and *DlFT2* genes are represented by numbers 1 to 3 and 16 to 18, respectively, with a scale of 1 cm. **e** The statistics of the number of rosette leaves during flowering for both wild-type and transgenic *Arabidopsis* plants, with six plants used for each line. The statistics for wild-type *Arabidopsis* began approximately 30 days after sowing, while the OE-*DlFT1* lines started around 26 days after sowing. The OE-*DlFT2* lines commences around 36 days after sowing. The data is presented as mean ± SD. One-way analysis of variance (ANOVA) was performed. *P* value: *P < 0.05, **P < 0.01, ***P < 0.001

Florigen FT induces flowering in angiosperms, but some FT homologs repress flowering (Wickland et al. 2015). A previous study demonstrated that the antagonistic function of FT homologs is caused by changes in the external loop of the FT protein in sugar beets, which is encoded by segment B of the fourth exon (Pin et al. 2010). To identify the potential flowering inducer and repressor of FT homologs in Sapindaceae, the FT amino acid sequences of the segment B fragments from the six Sapindaceae species and other functionally characterized species were aligned (Fig. 2a). Tryptophan at position 138 (W138) was conserved across Sapindaceae species. This observation suggests that the amino acid substitution in position 134, either a Y (tyrosine) or a non-Y residue, may be more important for the functional divergence of FT-like homologs in Sapindaceae species (Fig. 2a). Based on the amino acid variation in position 134, we found that there are two types of *FT* gene in Sapindoideae, Y in FT1/FT3/FT4-like and N in FT2-like (Fig. 2a). Subsequently, we performed a folding prediction and our analysis revealed that the external loops in segment B of longan FT1 (the main floral inducer) and FT2 were conformationally different (Fig. 2b). This finding suggests that differences, particularly at residue Y134/N134, may confer functional antagonism between these two genes. We then detected the changes in expression of *DlFT1* and *DlFT2* in the shoot apices of longan before the initiation of floral primordia. The results revealed that the expression levels of *DlFT1* and *DlFT2* were nearly absent (Fig. S3a), suggesting that *FT* in longan is barely expressed in the shoot apices. We further explored the expression profiles of *DlFT1* and *DlFT2* in leaves during natural flowering, and found that a dramatic increase in *DlFT1* from − 28 to − 7 days before flowering, while *DlFT2* exhibited a slight decrease (Fig. 2c). Additionally, the expression of *LcFT1* in lychee was enhanced during reactive oxygen species (ROS) and low-temperature treatments to induce flowering, while *LcFT2* decreased expression in leaves (Lu et al. 2020a; Zhang et al. 2017) (Fig. S3c, d), suggesting their possible functional antagonism. Taken together, we infer that the *FT1*-like genes may be conserved flowering inducers in Sapindaceae, while the *FT2*-like genes may function as flowering repressors due to the key substitution at FT residue 134.

To validate whether *FT1* and *FT2* promote or inhibit floral transition among Sapindaceae species, *DlFT1* and *DlFT2* were constitutively overexpressed and driven by the CaMV 35S promoter in *Arabidopsis*. Under long-day conditions, *DlFT1* overexpression plants started to flower when there were 7.5 rosette leaves, much earlier than WT control which flowered with 14.3 leaves (Fig. 2d, e, Fig. S3b). Conversely, the floral initiation of *DlFT2* transgenic plants was dramatically delayed until bearing 31.0 leaves (Fig. 2d and e**,** Fig. S3b). Thus, ectopic expression of *DlFT1* dramatically accelerated floral initiation, while ectopic expression of *DlFT2* substantially postponed floral initiation, confirming their functional antagonism in flowering induction.

### Specific insertion in the *DlFT1* promoter may be involved in flowering diversification in longan

**Fig. 3:**
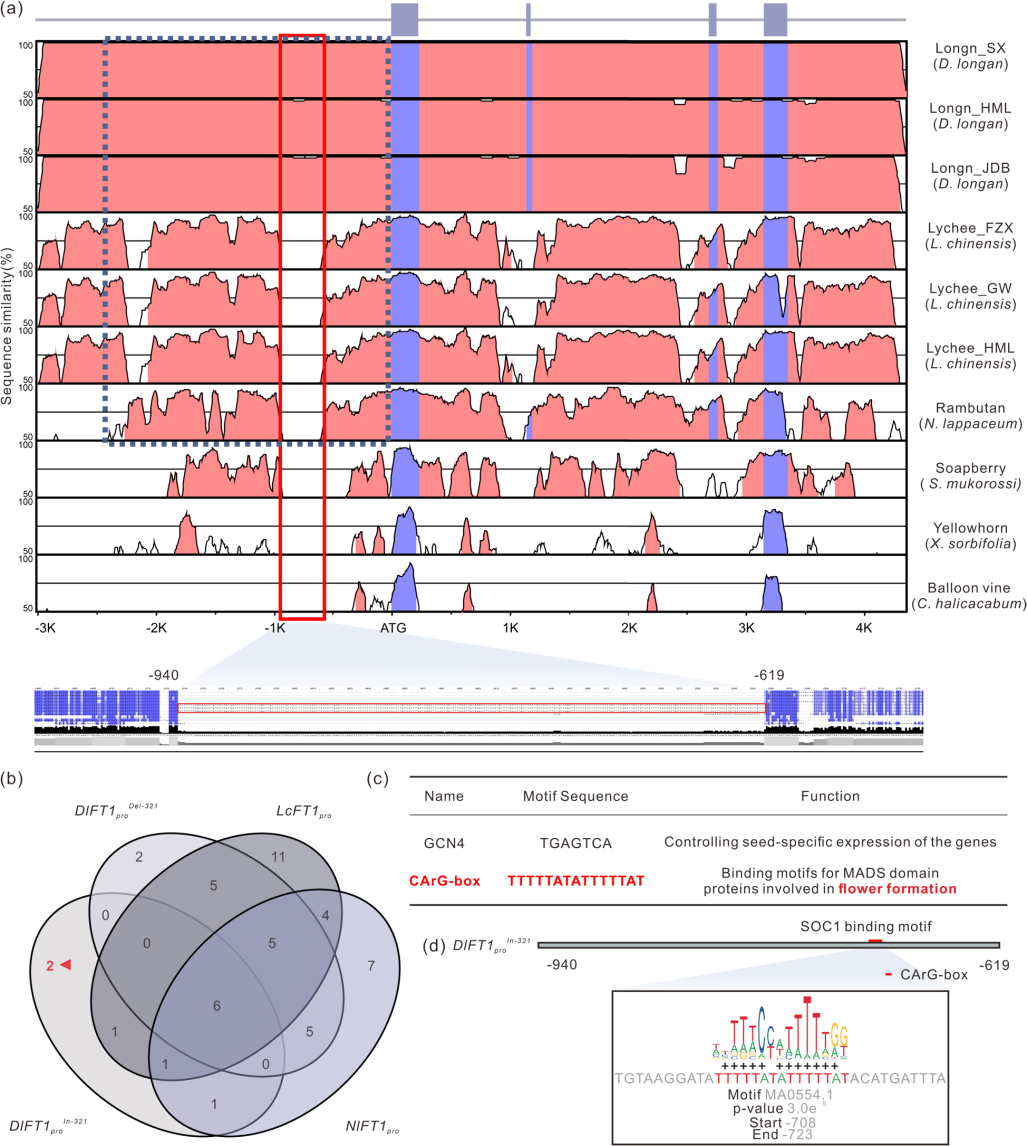
Conservation analysis of *FT1*-like promoter sequences in Sapindaceae species. **a** Pairwise alignment of *FT1* nucleotide sequences linked to upstream of the ATG, spanning 3.0 kilobases, and downstream of stop codons, spanning 1.0 kilobase from six Sapindaceae species by using mVISTA (Brudno et al. 2003). The graphical output displays the base pair identity within a sliding window of 75-bp, ranging from 50 to 100%. The upper shows the *FT1*-like gene structure and the bottom with red box shows longan-specific conserved segments in the multiple sequence alignment of different Sapindaceae species by ClustalW2 (Larkin et al. 2007). The approximately 2.5-kb upstream sequence of the ATG in lychee, longan, and rambutan showed a high degree of conservation, marked by blue dotted box (**b**) Venn diagram assessing the count of CREs disparately among *DlFT1*_*pro*_^*Del−321*^ (2.5-kb upstream from the ATG of *DlFT1* out of longan-specific 321-bp insertion), *DlFT1*_*pro*_^*In−321*^(longan-specific 321-bp insertion located between 619 and 940 nucleotides upstream from the ATG of *DlFT1*), *LcFT1pro* (2.5-kb upstream from the ATG of *LcFT1*), *NlFT1*_*pro*_ (2.5-kb upstream from the ATG of *NlFT1-a*). The red triangle highlights the unique CREs of longan-specific conserved segments in *DlFT1*_*pro*_^*Del−321*^_,_ *DlFT1*_*pro*_^*In−321*^, *LcFT1*_*pro*_, and *NlFT1*_*pro*_. **c** *Cis*-regulatory sequences of longan-specific 321-bp insertion are highlighted in b with red triangle. **d** *Cis*-element prediction on the longan-specific 321-bp insertion and red rectangle highlight the TF binding motif (TFBM) of SOC1 using MAST (Timothy et al. 1998), by which sequences with an *E*-value less than 10 are included in the output. The motif logo derived from JASPAR TF binding profile associated with SOC1(MA0054.1) is provided in bottom black box

As *FT1*-like genes are the flowering-promoting genes in Sapindaceae, we next studied the regulatory differences in the *FT1*-like genes among the Sapindaceae species. Nucleotide sequences of the *FT1*-like genes (including 3.0 kilobases (kb) upstream and 1.0 kb downstream regions) were extracted from three longan varieties (‘SX’, ‘HML’, and ‘JDB’), three lychee varieties (‘FZX’, ‘GW’, and ‘HML’), as well as rambutan, soapberry, yellowhorn, and balloon vine for comparative analysis (Adrian et al. 2010). Interestingly, the alignment revealed the specific 321-bp insertion located between 619 and 940 nucleotides upstream from the *DlFT1* start codon in the three longan cultivars (Fig. 3a, highlighted in red box). To investigate whether this 321-bp insertion harbored longan-specific CREs, we compared the CREs among the complete promoter regions of *LcFT1*, *NlFT1*, the 321-bp insertion, and the *DlFT1* promoter region and excluding the 321-bp insertion (Fig. 3b). Consequently, two specific CREs within the 321-bp insertion of the *DlFT1* promoter were identified (Fig. 3b, Table S3, Xu et al. 2011; Yin et al. 2016). One was an SRF-type CArG-box (CC[A/T]6GG) (Fig. 3c), which is a binding motif for the MADS domain proteins associated with flower formation (Tao et al. 2012). For example, the MADS-box protein SOC1, which activates FT transcription by directly binding to the CArG motif within its promoter (Kou et al. 2022), has the potential to bind to the SRF-type CArG-box, suggesting that a specific regulation pathway may be involved in flowering of longan (Fig. 3d).

## Promoter diversity of tandemly duplicated *NlFT1s* likely contributes to their subfunctionalization in rambutan

**Fig. 4:**
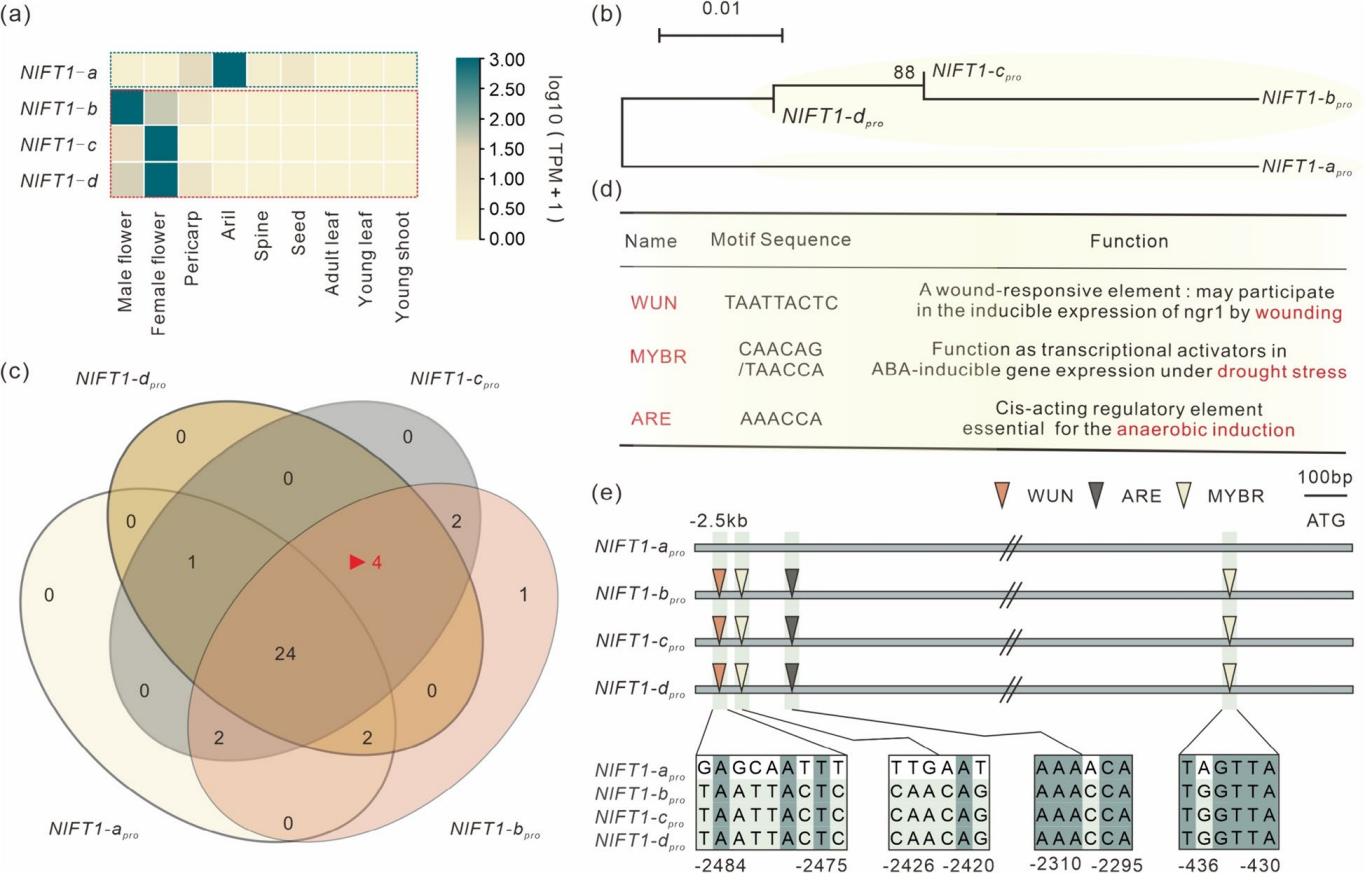
Scanning the promoter sequence of *FT1s* in rambutan. **a** The gene expression patterns of *FT*-like genes across various tissues in rambutan. **b** A Neighbor Joining phylogenetic analysis of promoter sequence associated with 4 *NlFT1s* in rambutan. **c** Venn diagram compares the number of CREs differentially among 4 *NlFT1s* linked to promoter sequence. The red triangles indicate the count of CREs in the promoter regions of *NlFT1-b*, *NlFT1-c*, and *NlFT1-d* that are different from those in *NlFT1-a*. **d** The promoter regions of *NlFT1-b*, *NlFT1-c*, and *NlFT1-d* contain identical CREs that are distinct from those present in the *NlFT1*-a gene. **e** Identification of CREs in the promoter regions of *NlFT1s*. CREs are depicted as triangles with different colors. The box displays the sequence of each CRE position in the four *NlFT1* genes. In the box there displayed the forward (WUN/MYB/ARE) or reverse complementary motif (One of the two MYB on the right) sequences within *NlFT1* gene promoter regions

We characterized four tandemly replicated *FT1*-like paralogs in rambutan (Fig. 1c). To explore whether these *NlFT1s* are functionally different, we analyzed their coding sequences and determined that *NlFT1-a* shared 95.98%, 94.25%, and 94.25% amino acid identity with *NlFT1-b*, *NlFT1-c*, and *NlFT1-d*, respectively (Fig. S4a), suggesting that their functions are likely similar. Additionally, we investigated the expression patterns across various tissues and noted that *NlFT1-a* showed a distinct expression pattern while the remaining three *NlFT1* shared similar expression profiles, with their main expression occurring in flowers (Fig. 4a, Fig. S4c). We inferred that the differential gene expression pattern between *NlFT1-a* and *NlFT1-b*/*c*/*d* was due to differences in the promoter region. Therefore, we employed the phylogenetic shadowing method to investigate the similarity in the *NlFT1-a* and *NlFT1-b*/*c*/*d* promoter regions. As results, the *NlFT1-b*/*c*/*d* promoter region showed similar changes compared with *NlFT1-a* (Fig. S4b). The evolutionary phylogeny analysis revealed that the *NlFT1-a* promoter sequence was different from the other *NlFT1s*, and the *NlFT1-b*, *NlFT1-c*, and *NlFT1-d* promoter regions shared higher sequence similarity (Fig. 4b). Thus, *NlFT1* genes may be subfunctionalized into two subgroups.

To investigate the variations between the *NlFT1-a* and *NlFT1-b*/*c*/*d* promoters, the *cis*-elements in the 2.5-kb *NlFT1-a*/*b*/*c*/*d* promoter sequences were analyzed. In total, 29, 35, 33, and 31 types of CREs were predicted from *NlFT1-a*, *NlFT1-b*, *NlFT1-c*, and *NlFT1-d*, respectively (Fig. 4c). The *NlFT1-b*/*c*/*d* promoter sequences shared four identical CREs (targeted by WUN, two MYBs, and ARE) that were absent in the *NlFT1*-a promoter region. All of these shared CREs in *NlFT1-b*/*c*/*d* were stress-response relative regulatory elements (Fig. 4d, e, Table S4, Xu et al. 2011; Yin et al. 2016), suggesting that *NlFT1-b*/*c*/*d* may adopt additional regulation in response to abiotic stress.

### SNP in the *LcFT1* promoter affects flowering time by influencing the binding affinity of SVP proteins

**Fig. 5:**
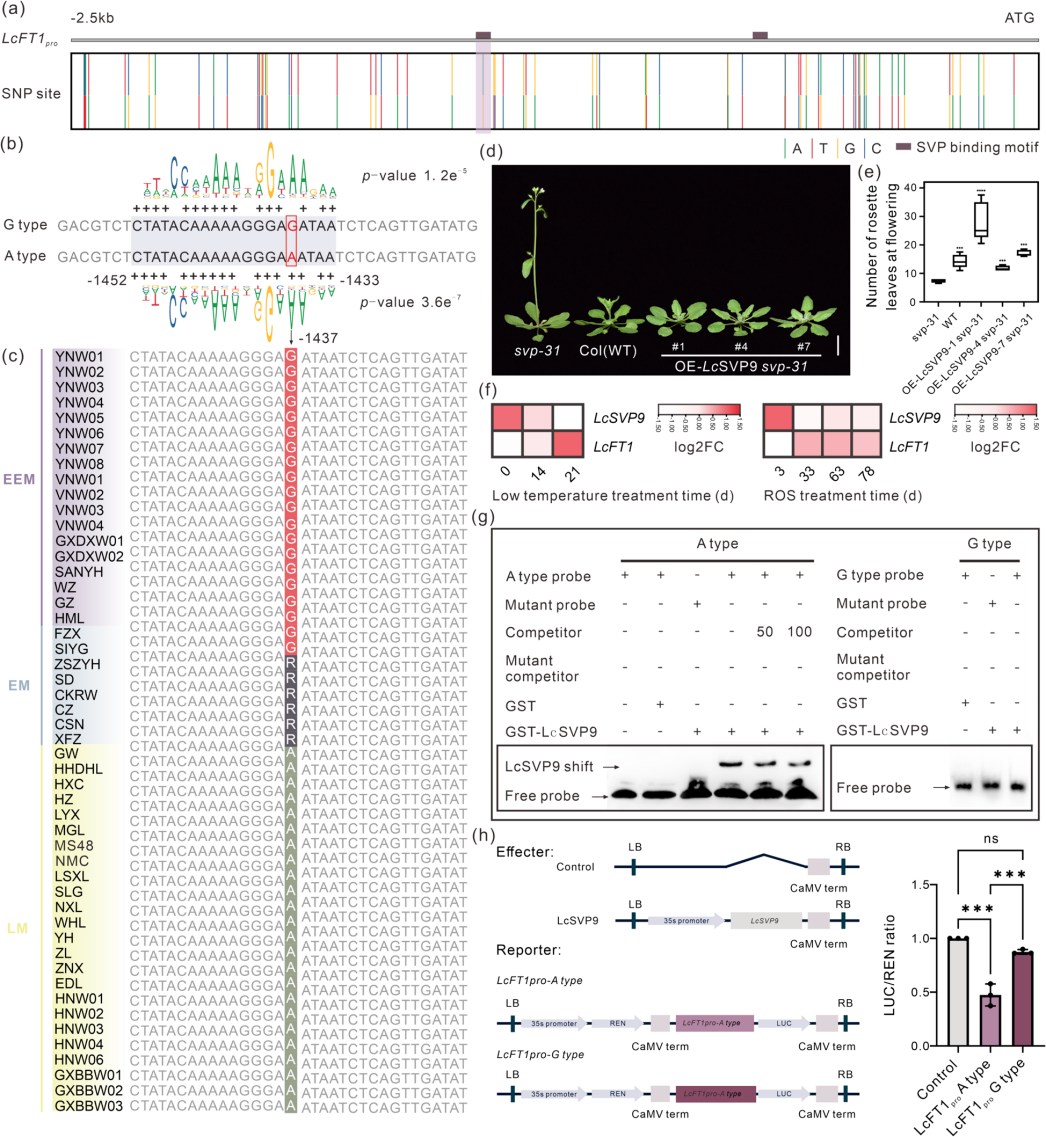
SNP-1,437 affected the transcriptional regulation of LcSVP9 on *LcFT1.* **a** Distribution of SNP in *LcFT1* promoter sequence of 47 lychee germplasms. The dark purple square exhibits the binding motif of SVP protein predicted by MAST. **b** The specific binding sites of SVP in two genotypes were displayed, and the *p*-value indicated the comparison of their binding ability. Motif logo derived from JASPAR TF binding profile associated with SVP (MA0555.1) is provided above and below of the gray background, respectively. **c** Homozygous and heterozygous SNPs from each accession in SVP binding site. The red background G illustrates the G homozygous genotype at the SVP-bound SNP site, while the green background A represents the A homozygous genotype. The gray background R shows the G/A heterozygous genotype. EEM is short for "extremely early maturing."; EM is short for "early maturing."; LM is short for "late maturing." **d** The phenotype of *Arabidopsis* with the overexpression of lychee *LcSVP9* genes. All plants were grown under long-day conditions with 16 h of light and 8 h of darkness at 23℃. The image depicts the phenotype after 24 days of sowing. Col (WT) represents the wild-type *Arabidopsis* with a Col-0 background. OE represents the transgenic lines with *LcSVP9* overexpression. T_2_ generation lines with *LcSVP9* gene are represented by numbers 1, 4 and 7, with a scale of 1 cm. **e** The statistics of the number of rosette leaves during flowering for *svp-31* mutants, wild-type and *LcSVP9* transgenic *Arabidopsis* plants, with five plants used for each line. The statistical analysis for *svp-31* mutants commenced around 24 days post-sowing, while wild-type *Arabidopsis* started approximately 27 days after sowing, and the OE-*LcSVP9* lines began between 28 and 42 days after sowing. The data is presented as mean ± SD. One-way analysis of variance (ANOVA) was performed. *P* value: ***P < 0.001, ****P < 0.0001. **f** Expression levels of *LcSVP9* and *LcFT1* in response to ROS treatment (Lu et al. 2020a) and low temperature (Zhang et al. 2017) during floral induction according to the RNA-seq data in leaves of lychee. TPM (transcripts per million) was used to indicate gene expression levels or transcript accumulated levels. The log_2_ ratio of fold change (log2FC) of the gene expression value between control and treatment was calculated according to the treatment timepoint. **g** EMSA was performed to compare the binding affinity of LcSVP9 in the promoter region of *LcFT1*^*A type*^ and *LcFT1*.^*G type*^ containing the SNP-1,437. **h** Transient dual-luciferase expression assay. The control group included an empty vector, and the data are presented as the mean ± SD derived from three biologically independent samples (black dots). One-way ANOVA* p* value: ns = 0.097, ***P < 0.001b

After comparing the promoter region of *FT1*-like genes among the Sapindaceae species and different *FT1* loci in rambutan, we determined that some *FT1*-like genes may have been subfunctionalized by the evolution of their promoter sequences. Then, we examined whether the change in a single locus of the promoter region could contribute to the regulatory diversity of the *FT1* gene within a single species. Based on previously published resequencing data from 47 lychee germplasms (Hu et al. 2022), 67 SNPs were detected in the *LcFT1* promoter sequences (Fig. 5a). We predicted two SVP binding motifs (intermediate type CArG box) in the highly conserved 2.5-kb sequence of the *LcFT1* promoter (Fig. 5a). Notably, we discovered a SNP situated within one of these binding motifs, which was widely shared across 47 distinct lychee varieties (Fig. 5b, c, Table S5). The consensus sequence of the binding motif (CTATACAAAAAGGGA(G/A)ATAA) was located from 1,452 to 1,433 nucleotides upstream from the *LcFT1* start codon, with a G/A SNP positioned at 1,437-bp (Fig. 5b). Interestingly, almost all of the extremely early-maturing lychee varieties (see purple shadow in Fig. 5c) exhibited the G allele, whereas the early-ripening lychee varieties (see blue shadow in Fig. 5c) possessed both genotypes (R represents a heterozygous G/A genotype). In contrast, all late-ripening lychee varieties (see yellow shadow in Fig. 5c) exclusively exhibited the A genotype (Fig. 5c). According to the single binding site prediction using position weight matrix (PWM) scanning, the A-type motif had a higher binding affinity to SVP than the G-type motif (Fig. 5b).

To confirm whether the G/A SNP affects the binding affinity of the SVP protein, we assessed the binding ability of these two types of motifs compared to lychee SVP. In total, there were ten *SVP* homologs in the lychee genome. Among them, *LcSVP9* belongs to the *SVP1* clade, which is associated with delayed flowering (Fig. S6, Liu et al. 2018) and exhibits preferential expression in lychee leaves (Fig. S5) similar to the *Arabidopsis SVP* (Hartmann et al. 2000). Thus, we presume that *LcSVP9* may function similarly to *AtSVP* in delaying the flowering time of plants. Also, *LcSVP9* exhibited the highest expression level in lychee leaves among these ten *SVP* genes (Fig. S5a). We hypothesize that LcSVP9 could be a key regulator within the *SVP* gene family. To confirm the function of LcSVP9 in regulating flowering, we introduced the *LcSVP9* gene into *svp-31* mutant *Arabidopsis* under the control of the CaMV 35S promoter. Under long-day conditions, plants overexpressing *LcSVP9* initiated flowering with 11.8 to 28.6 rosette leaves, showing a significantly delayed flowering time compared with the s*vp-31* mutant control (7.4 rosette leaves) and a timing similar to the WT control (14.3 rosette leaves) (Fig. 5d, e, Fig. S5c). This suggests that the expression of *LcSVP9* impacted flowering time, functioning as a floral repressor like *AtSVP*. We attempted to determine the expression levels of known flowering time genes in *LcSVP9* overexpression plants. We found that the expression level of *AtFT* were significantly reduced, indicating that *LcSVP9* regulates flowering via repressing *AtFT* (Fig. S5d). This finding is consistent with the observation that levels of *AtFT* expression were elevated in *svp-31* mutants (Fig. S5d).

Furthermore, we observed a reduction in *LcSVP9* levels in leaves subjected to reactive oxygen species (ROS) and low-temperature treatments, while *LcFT1* exhibited contrasting expression patterns (Fig. 5f). Thus, we propose that LcSVP9 may repress the expression of *LcFT1*. Initially, we performed an electrophoretic mobility shift assay (EMSA) to investigate the potential impact of the − 1,437 SNP on the binding affinity between LcSVP9 and the *LcFT1* promoter. The results showed that the LcSVP9 recombinant protein bound to the A-type motif but did not bind to the G-type motif (Fig. 5g), confirming that the natural variation of nucleotide position − 1,437 in the *LcFT1* promoter are correlated with differential binding of LcSVP9. Subsequently, we conducted a dual-luciferase reporter assay to investigate the transcriptional activity of LcSVP9 on *LcFT1*^*A−type*^ and *LcFT1*^*G−type*^ promoters. The results showed LcSVP9 significantly reduced the expression of the LUC reporter by interacting with *LcFT1*^*A−type*^ promoter (Fig. 5h). In contrast, LcSVP9 lost its transcriptional repression on LUC driven by the *LcFT1*^*G−type*^ promoter (Fig. 5h). Therefore, the presence of the SVP-binding motif of *LcFT1*^*A−type*^ promoters is crucial for the inhibition of *LcFT1* expression by LcSVP9. We then analyzed data from ten lychee leaf samples collected in the first half of December 2014, sourced from public transcriptome data (Lu et al. 2022). The results indicated that the expression of *LcFT1* was significantly higher in the early-maturing variety (EM) compared to the late-maturing variety (LM) during the flowering induction period in most lychee (Fig. 5Sb). These findings are consistent with our initial expectations. The collective data suggest that the natural variation at nucleotide position − 1,437 of the lychee *FT1* promoter influences the binding affinity of LcSVP9, a negative regulator, thereby leading to differential expression of *LcFT1*, which is likely implicated in regulating lychee flowering time.

## Discussion

The number of PEBP genes varied greatly across the Sapindaceae species, ranging from nine to twelve copies (Fig. 1b). Most eudicot species typically have fewer than ten PEBP genes, with a few exceptions, including turnips (*Brassica rapa*), soybean (*Glycine max*)*,* tomato (*Solanum lycopersicum*), and lotus (*Nelumbo nucifera*), where the number exceeds ten due to additional whole genome duplication (WGD) events (Liu et al. 2016. No further recent WGD events have been observed in Sapindaceae, with the exception of the two WGD events following the γ event that are shared among all dicot plants (Zheng et al. 2022; Xue et al. 2022; Hu et al. 2022; Wang et al. 2022). Therefore, local duplications are more noticeable in the expansion of the PEBP-family genes among the Sapindaceae genomes. Phylogenetic analysis indicated that *FT1*-like, *FT2*-like, and *FT3*-like were present in lychee, longan, rambutan, and soapberry, suggesting that duplication occurred before the species diverged (Fig. 1a). Despite that the genes located upstream and downstream of *FT2*-like were highly collinear across all six Sapindaceae species, a homologous *FT2* gene was not identified in yellowhorn. This observation indicates that the FT2-like clade may be traced back to a common ancestor in the subfamily Sapindoideae, which encompasses the other five Sapindaceae species, excluding yellowhorn, which is in the subfamily Dodonaeoideae. No FT2-like flowering inhibitory factor has been identified in yellowhorn (Fig. 2a), which may be linked to the absence of juvenile phase characteristics in this species (Yao et al. 2013). The *MFT*-like gene was amplified in balloon vine compared to the other five Sapindaceae species (Fig. 1b). Gene collinearity analysis showed that balloon vine *MFT1*-like was arranged in tandem with six copies (Fig. S2a). The expression analysis of the six tandemly duplicated *CaMF1* genes demonstrated a predominant expression pattern in the seeds (Fig. S2b). Previous studies have reported that the *CaMF1* homolog *MFT* is involved in promoting seed germination (Danilevskaya et al. 2008). Thus, we hypothesized that the function of CaMF1 in balloon vine may also be associated with seed germination, specifically the ability of balloon vine seeds to maintain their vitality and germination capacity, even after prolonged storage, in contrast to the limited preservation capacity of lychee and longan seeds (Zhu et al. 2019; Johnston et al. 1979). This distinct characteristic is likely attributed to the amplification of *CaMFT1*.

A comparative analysis revealed that the 321-bp insertion in the longan promoter region, which was absent in the closely related species lychee and rambutan, contained an SRF-type CArG-box domain which would be bound by SOC1 (Fig. 3d). Furthermore, previous research has suggested that SOC1 functions as a transcriptional activator of *FT* in soybean leaves by directly binding to the CArG motif within the *FT* promoter (Kou et al. 2022). We speculate that SOC1 regulates *DlFT1* expression in longan leaves by binding to the CArG box on the *DlFT1* promoter within the 321-bp insertion specific to longan, thereby controlling flowering time. Longan exhibits unique floral induction in response to KClO_3_ application, a capability unparalleled in other Sapindaceae species (Matsumoto et al. 2007). Therefore, we speculate that SOC1 may respond to KClO_3_ treatment and bind to a species-specific motif in the *DlFT1* promoter, accelerating the expression of *DlFT1* to promote the transition to reproductive development and flowering. Further analysis should be performed to validate this possibility.

The *NlFT1* gene undergoes tandem duplication in rambutan, resulting in four copies (Fig. 1c). Their regulatory sequences and expression patterns in different biological processes were in two distinct branches and one *NlFT1-a* clade, and the other three genes *(NlFT1-b*, *NlFT1-c*, and *NlFT1-d*) clustered in a single clade (Fig. 4a, b, Fig. S4c). This result suggests that the expression of the *NlFT1-b*, *NlFT1-c*, and *NlFT1-d* genes may be regulated by common CREs. We found that *NlFT1-b*, *NlFT1-c*, and *NlFT1*-d contained three types of identical CREs that are distinct from those found in the *NlFT1-a* gene. These CREs were associated with stress responses, including the WUN motif for wound responsiveness, the MYB-target motifs, and the anaerobic response (Fig. 4c, d). A previous study showed that the WUN-motif elements are bound by NAC transcription factors (Huang et al. 2017). In response to drought, the NAC-type transcription factor VASCULAR PLANT ONE-ZINC FINGER 1 (SlVOZ1) directly binds to the promoter of the major flowering-integrator gene *SINGLE FLOWER TRUSS* (*SFT*), an FT orthologue, thereby promoting the transition to flowering in tomato (Chong et al. 2022). Thus, we speculate that the three rambutan *NlFT1* genes (*NlFT1-b*, *NlFT1-c,* and *NlFT1-d*), resulting from gene replication, regulate flowering through stress response pathways.

A comparative analysis of the regulatory regions was conducted to investigate inter-specific variations in different lychee germplasms. Variations in the CREs were identified within the regulatory regions of the *FT1*-like gene in lychee. Our experiments showed that natural variation occurring at position -1,437 within the *LcFT1* promoter correlates with differential binding of LcSVP9 transcription factors, potentially influencing lychee flowering time. This variation in the CREs offers a potential molecular marker for lychee breeding. Modifications in CREs are linked to alterations in diverse agronomic traits (Rodríguez-Leal et al. 2017; Hendelman et al. 2021). For example, a recent study utilized *cis*-regulatory editing to alter the transcription of WOX9 in tomatoes, which subsequently impacted floral development and mitigated undesired outcomes (Hendelman et al. 2021). The recent application of CRISPR/Cas9-mediated genome editing technology to improve lychee varieties (Wang et al. 2023a) offers promising possibilities for biotechnological engineering of Sapindaceae species. For example, by modifying the regulatory *cis*-element that controls *FT-*like gene expression, the flowering period and the period of fresh fruit supply in commercially important fruit trees in Sapindaceae can be extended.

## Materials and methods

### Plant materials

This study utilized eight-year-old Dimocarpus longan cv. Shixia trees. The experimental trees were cultivated at South China Agricultural University in Guangzhou, China (lat. 23.1568° N, lon. 113.3537° E). The terminal shoots of three trees were sampled to obtain adult leaves, with three biological replicates collected for each sample.

### Identification of PEBP family members from six *Sapindaceae* species

The conserved domain (PF01161) of the PEBP was acquired fromtp://pfam.xfam.org/ (Mistry et al. 2021; Wang et al. 2023b). TBtools (Chen et al. 2020) was utilized to perform HMMER analysis, allowing for the retrieval of protein data and the identification of potential members of the PEBP family. The identified genes were screened and validated using two online tools: Ptps://www.ebi.ac.uk/Tools/pfa/pfamscan/) (Madeira et al. 2019) and NCBI Conserved tps://www.ncbi.nlm.nih.gov/Structure/cdd/wrpsb.cgi) (Lu et al. 2020b)_._ Genes without complete PEBP domains were eliminated for further analysis.

### Phylogenetic analysis and multiple sequence alignment

The PEBP protein sequences in other 46 seed plants species characterized by transgenic approaches, were retrieved from the Phytozome, GenBank and Arabidopsis Information Resource (TAIR) database (Table S2). A Neighbor Joining phylogenetic analysis was conducted on a total of 156 PEBP homologs across 52 flowering plants in MEGA X (V.10.2.6) (Kumar et al. 2018). The clades were assigned based on the observed clustering pattern among genes in Arabidopsis. The Taxonomy tool (Schoch et al. 2020) avaitps://www.ncbi.nlm.nih.gov/ was utilized to conduct a species phylogenetic tree. Pairwise alignments of *FT1*-like promoter sequences from the six Sapindaceae species were created using mVISTA Shuffl://genome.lbl.gov/vista) (Brudno et al. 2003). The conserved regions were aligned using ClustalW2 (V.2.1) (Larkin et al. 2007).

### Synteny analysis among six *Sapindaceae* plants

Syntenic gene pairs were identified among six plants from the Sapindaceae fami-ly using JCVI://github.com/tanghaibao/jcvi/wiki/MCscan-(Python-version))(Tang et al. 2008). The identification of syntenic blocks for each pair of species was performed usi-ng the 'jcvi.compara.catalog ortholog –cscore = 0.7' parameter.

### Analysis of CREs in the lychee, longan, and rambutan

The promoter sequence of the *FT1*-like gene was obtained by extracting the 2500-bp sequence upstream of the translation initiation site. To identify potential *cis*-regul-atory elements, promoter sequences were predicted using Plformatics.ps-b.ugent.be/webtools/plantcare/html/) (Lescot et al. 2002) aps://meme-suite.org/meme/tool-s/mast) (Timothy et al. 1998), by which sequences with an *E*-value less than 10 were included in the output. The motif profile of SVP protein w-as derived from://jaspar.genereg.net//) (Wasserman et al. 2004).

### RNA-Seq data analysis

All raw pair-ends reads were trimmed utilizing trimmomatic (V.0.36) (Bolger et al. 2014) to remove the adapters and low-quality bases. Subsequently, cleaned reads were aligned to the reference genomes using the STAR alignment tool (V.2.7.10b) (Dobin et al. 2013). The read counts and Transcripts Per Million Reads (TPM) for the genes were computed using the StringTie (V.2.2.1) (Kovaka et al. 2019)_._

### Folding prediction for the DlFT1 and DlFT2 proteins

The DeepView/Swiss-pdb viewer (V.4.0.1) (Schwede et al. 2003) was used to predict the structure of DlFT1 and DlFT2 proteins, based on the Arabidopsis FT structure (PDB ID: 1WKP). The three-dimensional protein structures of DIFT1 and DIFT2 were obtained by utilizing the SWISwissmodel.expasy.org) (Waterhouse et al. 2018).

### Constructs for transgenic plants

The coding sequences of *DlFT1, DlFT2* and *LcSVP9* were cloned and inserted into the pEarleyGate 201 vector, which harbored the CaMV 35S promoter. The recombinant plasmids pEarleyGate 201-*DlFT1*, pEarleyGate 201-*DlFT2* and pEarleyGate 201-*LcSVP9* were introduced into Agrobacterium tumefaciens strain GV3101. Plants were transformed through the floral dip method and subsequently screened for resistance to BASTA. The T-DNA insertion line *svp-31* (SALK_026551) was acquired from the Arabidopsis Biological Resource Center (ABRC) (Alonso et al. 2003).

### Quantitative gene expression analysis

TRIzol, a product from Thermo Fisher Scientific, was utilized for the isolation of total RNA from longan leaves. TransScript® One-Step gDNA Removal and cDNA Synthesis SuperMix (TransGen, AT311-02) was utilized to synthesize cDNA from RNA samples with an amount of 0.5 μg. The qRT-PCR primers were synthesized by Sangon Co. Ltd. (Shanghai, China), using Primer 5.0 (Singh et al. 1998) for their design. The 2 × GoTaq® qPCR Master Mix from Promega was utilized for conducting the qRT-PCR experiment. The − delt CT and 2ˆ (− delta delta CT) calculation was employed to normalize expression levels. The primer sequences utilized can be found in Table S8.

### Dual-luciferase reporter assay

The coding sequence of LcSVP9 was cloned and inserted into the pGreenII-62-SK vector, serving as the effector. The pGreen II-0800-Luc vector was utilized to inserted the promoter fragments of *LcFT1*^*A−type*^ and *LcFT1*^*G−type*^ (Fig. 5e), which were subsequently employed as reporters. The empty vector served as the negative control. The effector and reporter plasmids were separately transformed into Agrobacterium strains GV3101 and subsequently co-transfected into N. benthamiana leaves. The OD600 of the bacterial cultures was adjusted to 1.0, with an effector to reporter ratio of 9:1. Dual luciferase reporter gene assay kit (YEASEN) was employed to measure the relative levels of luciferase activity, in accordance with the instructions provided by the manufacturer.

### EMSA

The coding sequence of LcSVP9 was cloned and inserted into the pGEX4T-1 vector. The recombinant protein GST-LcSVP9 was purified in *Escherichia coli* with Glutathione and Nibeads. The oligonucleotide probes were biotin-labeled at the 3’ end. The competition analysis reactions were conducted with the unlabeled probes. EMSA experiments were conducted utilizing the LightShift Chemiluminescent EMSA Kit (Thermo Fisher, 20148X).

### Supplementary Information

Supplementary Material 1.

## Data Availability

All assemblies with annotations underlying this article were available in the SapBase dttp://www.sapindaceae.com/Download.html). Transcriptome data from three tissues (young shoot, young leaf, and adult leaf) of rambutan were downloaded fro-m the NCBI SRA database (SRR14560235, SRR14560276, SRR14560287). RNA-Seq data from lychee leaves exposed to reactive oxygen species (ROS) and low-temperature treatments, along with lychee leaf samples collected in early December 2014, were deposited in the NCBI BioProjects PRJNA1045234, PRJNA1045227, and PRJNA766599, respectively. The remaining RNA-Seq were available at the SapBase dttp://www.sapindaceae.com/Gene-Expression-V2/GeneExpression.html). The lychee variants VCF files were downloaded from the Mendeley dps://data.mendeley.com/datasets/v37bv5jt6g/1).

## Abbreviations

Bp: Base pair
CREs: *Cis*-Regulatory elements
EMSA: Electrophoretic mobility shift
*FT*: *Flower Locus T*
Kb: Kilobases
KClO_3_: Potassium chlorate
LUC: Firefly luciferase
*MFT*: *MOTHER OF FT AND TFL1*
NS: Not significant
OE: Overexpressing
PEBP: Phosphatidylethanolamine-binding protein
qRT-PCR: Quantitative real-time polymerase chain reaction
REN: Renilla luciferase
ROS: Reactive oxygen species
*SVP*: *SHORT VEGETATIVE PHASE*
*SOC1*: *SUPPRESSOR OF OVEREXPRESSION OF CONSTANS 1*
SNP: Single nucleotide polymorphism
SD: Standard deviation
TPM: Transcripts per million
*TFL1*: *TERMINAL FLOWER 1*
UTR: Untranslated Region
WGD: Whole genome duplication
