# Supplementary material for: Diversification of FT-like genes in the PEBP family contributes to the variation of flowering traits in Sapindaceae species
Source: Mol Hortic. 2024 Jul 16;4:28. doi: 10.1186/s43897-024-00104-4 (PMC11251392; doi:10.1186/s43897-024-00104-4)
Supplement: Supplementary file 1 — Supplementary Material 1. [file 43897_2024_104_MOESM1_ESM.docx]

## Supplementary information


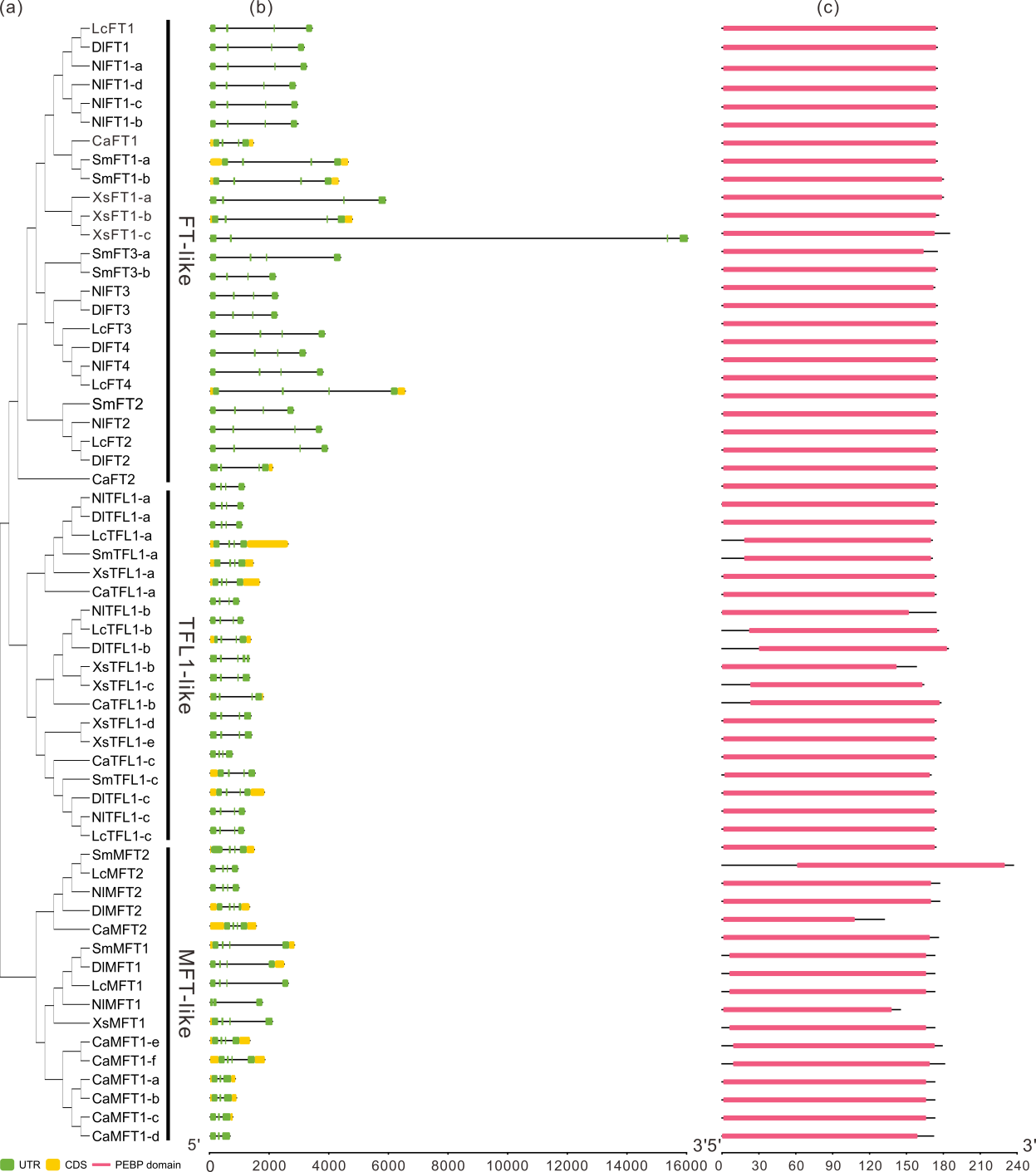


**Figure S1. Gene structures and domain of PEBP genes in six Sapindaceae species.**

(a) Phylogenetic tree of 60 PEBP protein sequences generated by using Neigbour-joining Tree method. (b) Gene structures of PEBP genes family members across 6 Sapindaceae species. Exons are represented by green boxes, introns are indicated by black lines, and UTRs are denoted by yellow boxes. (c) Analysis of sequence conservation in the PEBP protein domains across 60 PEBP genes.


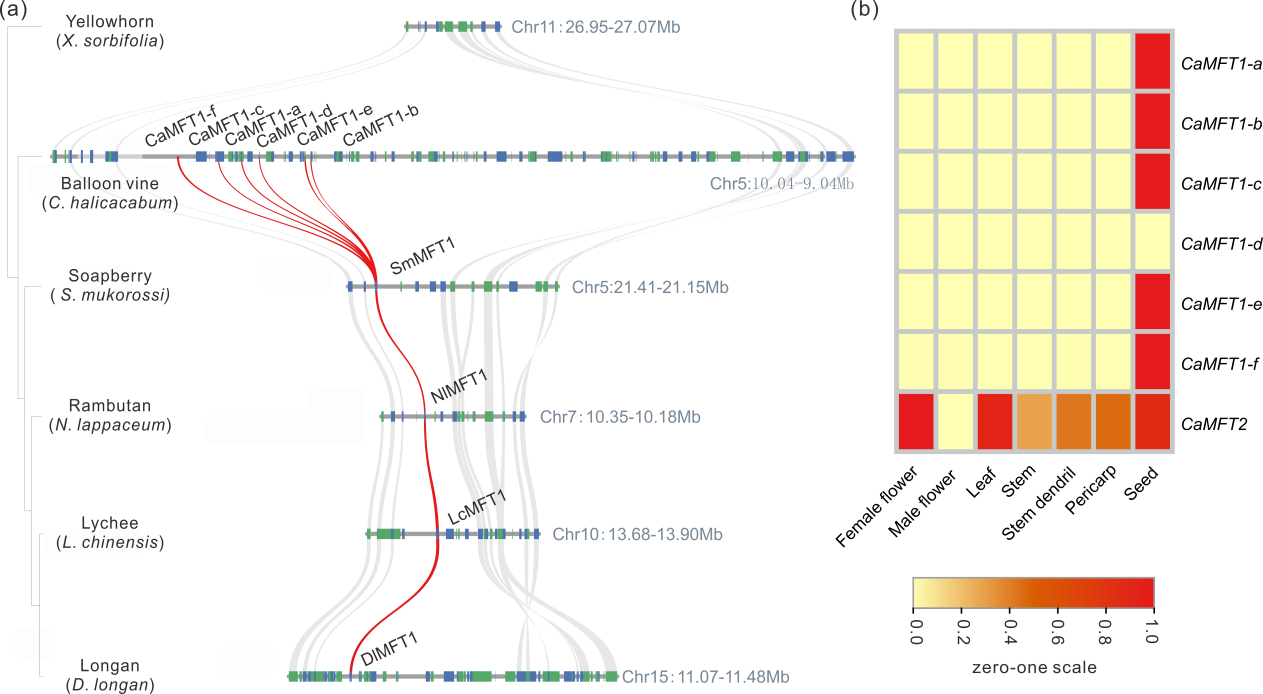


**Figure S2. The synteny of Balloon vine *MFT1* genes among 6 Sapindaceae species and the tissue-specific expression patterns of *MFT* genes in balloon vine.**

(a) The synteny across lychee, longan, rambutan, soapberry, yellowhorn and balloon vine of *MFT1* genes. The gray lines connect syntenic blocks. The *MFT1* gene pairs are linked with red curves. (b) The expression patterns of *MFT* genes across various tissues of the balloon vine.


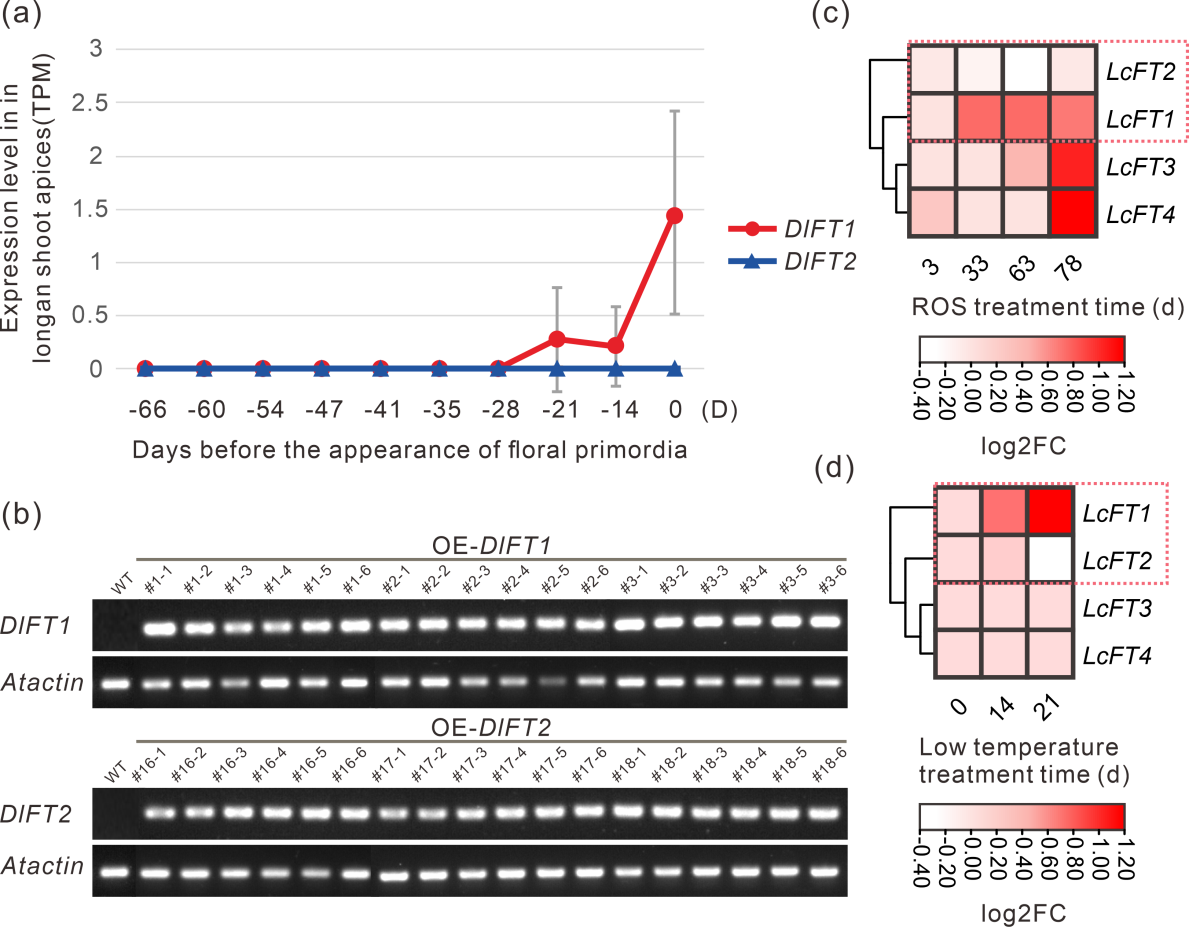


**Figure S3. Partial amino acid sequence alignment of PEBP family members and the expression profiles of *FT*-like genes.**

(a) The expression patterns of *DlFTs* during flower induction in longan shoot apices were analyzed through RNA-seq. (b) The semi-quantitative reverse transcription polymerase chain reaction (semi-qRT-PCR) was performed to detect overexpression in transgenic plants. (c) and (d) Expression profiles of 4 *LcFT*s in response to ROS treatment (Lu et al. 2020) and low temperature (Zhang et al. 2017) during floral induction according to the RNA-seq data in leaves of lychee. TPM was used to indicate gene expression levels or transcript accumulated levels. The log_2_ ratio of fold change (log2FC) of the gene expression value between control and treatment was calculated according to the treatment timepoint.


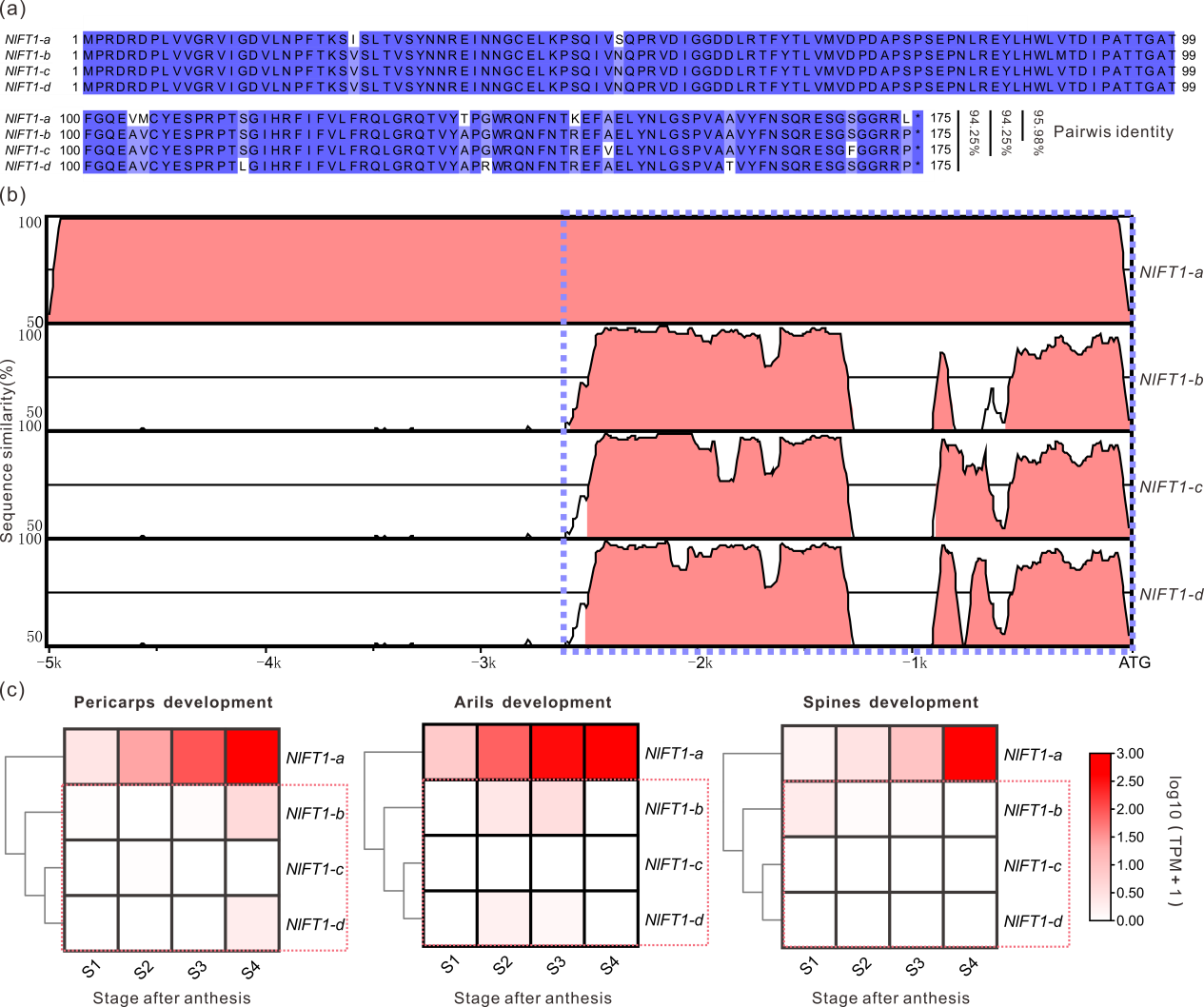


**Figure S4. The Sequence alignment of 4 *NlFT1s* in rambutan.**

(a) Amino acid identity comparisons of the protein sequences of the four *NlFT1s.* (b) Conservation analysis of Rambutan *FT1* Promoter Sequences. Pairwise alignment of *FT1* promoter sequences from four Rambutan *FT1* genes linked to upstream 5.0-kb using mVISTA (Brudno et al. 2003). The Graphical output displays the similarity of base pairs within a sliding window spanning 75 bp, ranging from 50 to 100%. The four conserved promoter sequences of rambutan *NlFT1* genes are demarcated with purple dashed lines. (c) Expression pattern of 4 *NlFT1s* genes during pericarps, arils, spines development, respectively.


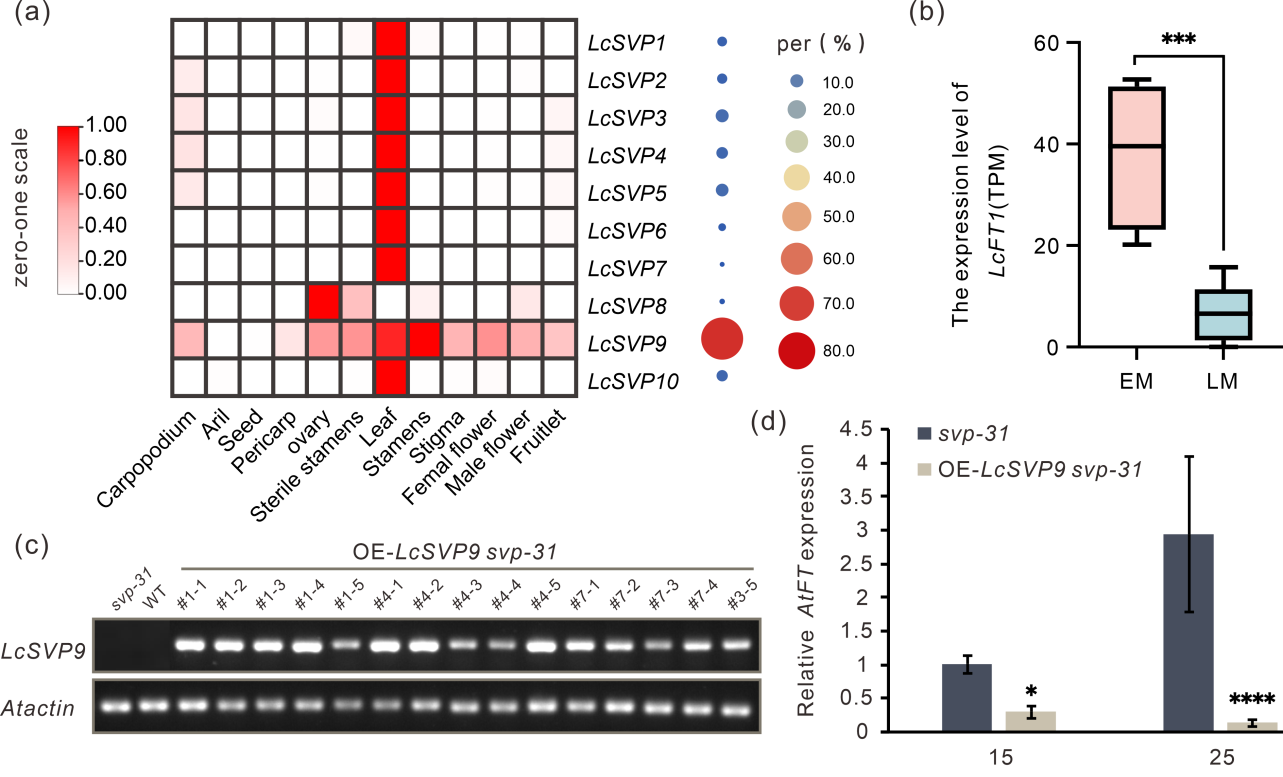


**Figure S5. The expression level of *LcSVPs and LcFT1* genes**

(a)The expression level of lychee SVP genes across different tissues. The size of the circles represents the expression proportion of *SVP* genes. (b) The expression levels of *LcFT1* in the leaves of different early-maturing (EM) lychee varieties, including four representative varieties ('FZX', 'CZ', 'CSN', and 'XFZ'), as well as late-maturing (LM) ones ('LYX', 'NMC', 'MGL', 'GW', 'HZ', and 'ZNX'). (c) The expression levels of *LcSVP9* in *LcSVP9* transgenic plants. (d) Expression of *AtFT* in leaves of long-day-grown *svp-31* mutants and *LcSVP9* overexpression plants during (day 15 and day 25) reproductive development.


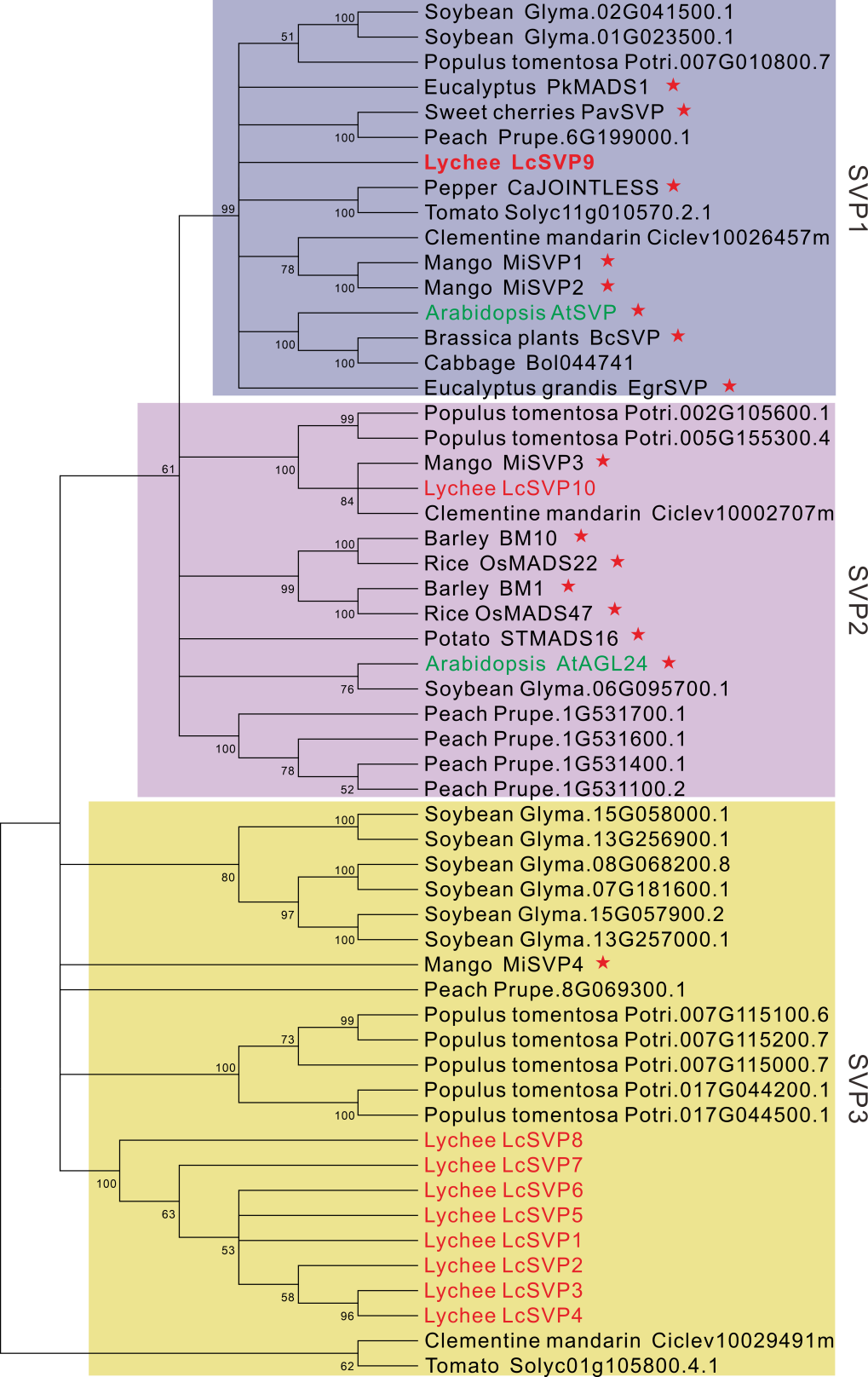


**Figure S6.** **Phylogenetic tree of typical *SVP* genes in eudicots.**

Group SVP1 in purple background; Group AGL24/SVP2 in light purple background; Group SVP3 in yellow background. Red star indicates floral transition function characterized by generating transgenic plants. Additionally, other *SVP* genes have been identified through research conducted by Liu et al (2018).

**Table S1. PEBP genes identified in six Sapindaceae species.**

| **Species** | **Gene ID** | **name** |
| --- | --- | --- |
| Lychee  (*Litchi chinensis*) | LITCHI002331.m1 | *LcFT1* |
|  | LITCHI002335.m1 | *LcFT2* |
|  | LITCHI002334.m1 | *LcFT3* |
|  | LITCHI006928.m1 | *LcFT4* |
|  | LITCHI009311.m1 | *LcTFL1-a* |
|  | LITCHI018838.m1 | *LcTFL1-c* |
|  | LITCHI024460.m1 | *LcTFL1-b* |
|  | LITCHI023105.m1 | *LcMFT1* |
|  | LITCHI029325.m1 | *LcMFT2* |
| Longan  (*Dimocarpus longan*) | Dil.05g001470.1.t1 | *DlFT1* |
|  | Dil.05g001570.1.t1 | *DlFT2* |
|  | Dil.05g001540.1.t1 | *DlFT3* |
|  | Dil.06g006560.1.t1 | *DlFT4* |
|  | Dil.08g015500.1.t1 | *DlTFL1-a* |
|  | Dil.13g008920.1.t1 | *DlTFL1-b* |
|  | Dil.14g009880.1.t1 | *DlTFL1-c* |
|  | Dil.15g013870.1.t1 | *DlMFT1* |
|  | Dil.07g017920.1.t1 | *DlMFT2* |
| Rambutan （*Nephelium lappaceum*） | Nl06g13640.mrna1 | *NlFT1-b* |
|  | Nl06g13650.mrna1 | *NlFT1-c* |
|  | Nl06g13660.mrna1 | *NlFT1-d* |
|  | Nl06g13670.mrna1 | *NlFT1-a* |
|  | Nl06g13690.mrna1 | *NlFT2* |
|  | Nl06g13680.mrna1 | *NlFT3* |
|  | Nl13g09360.mrna1 | *NlFT4* |
|  | Nl10g10020.mrna1 | *NlTFL1-a* |
|  | Nl15g07240.mrna1 | *NlTFL1-b* |
|  | Nl11g07990.mrna1 | *NlTFL1-c* |
|  | Nl16g04710.mrna1 | *NlMFT2* |
|  | Nla_Dil.15g013870.1.t1_1 | *NlMFT1* |
| Soapberry  （ *Sapindus mulorossi*） | whz_003057-RA | *SmFT1-a* |
|  | whz_003092-RA | *SmFT1-b* |
|  | whz_003088-RA | *SmFT3-a* |
|  | whz_003094-RA | *SmFT3-b* |
|  | whz_003087-RA | *SmFT2* |
|  | whz_008129-RA | *SmTFL1-a* |
|  | whz_026519-RA | *SmTFL1-c* |
|  | whz_012860-RA | *SmMFT1* |
|  | whz_002139-RA | *SmMFT2* |
| Yellowhorn  （*Xanthoceras sorbifolium*） | EVM0022396.1 | *XsFT1-a* |
|  | EVM0012685.1 | *XsFT1-b* |
|  | EVM0021328.1 | *XsFT1-c* |
|  | EVM0023266.1 | *XsTFL1-a* |
|  | EVM0000974.1 | *XsTFL1-b* |
|  | EVM0017724.1 | *XsTFL1-c* |
|  | EVM0019799.1 | *XsTFL1-d* |
|  | EVM0020336.1 | *XsTFL1-e* |
|  | EVM0005806.1 | *XsMFT1* |
| Balloon vine  (*Cardiospermum halicacabum*) | Ca01G06980.t1 | *CaFT1* |
|  | Ca01G06970.t1 | *CaFT2* |
|  | Ca04G10990.t1 | *CaTFL1-c* |
|  | Ca05G26900.t1 | *CaTFL1-a* |
|  | Ca02G09830.t1 | *CaTFL1-b* |
|  | Ca05G10880.t1 | *CaMFT1-a* |
|  | Ca05G10820.t1 | *CaMFT1-b* |
|  | Ca05G10900.t1 | *CaMFT1-c* |
|  | Ca05G10870.t1 | *CaMFT1-d* |
|  | Ca05G10830.t1 | *CaMFT1-e* |
|  | Ca05G10910.t1 | *CaMFT1-f* |
|  | Ca07G12370.t1 | *CaMFT2* |

**Table S2. Detailed information of PEBP genes from 46 seed plant species.**

| No. | **Species** | **Homologs** | **NCBI accession** |
| --- | --- | --- | --- |
| 1 | Alpine rock-cress (*Arabis alpina*) | AaTFL1-1 | BAK31016.1 |
|  |  | AaTFL1-2 | BAK31017.1 |
| 2 | Common bean (*Phaseolus vulgaris*) | PvTFL1y | ABR53777.2 |
| 3 | Barley (Hordeum vulgare) | HvFT1 | ADW82818.1 |
|  |  | HvFT2 | QOL02402.1 |
|  |  | HvFT3 | ABD75336.2 |
| 4 | Apple (*Malus domestica*) | MdTFL1 | NP_001280887.1 |
|  |  | MdTFL1-2 | NP_001280794.1 |
|  |  | MdFT2 | NP_001280810.1 |
|  |  | MdCENa | NP_001280813.1 |
|  |  | MdCENb | NP_001280940.1 |
| 5 | Potato (*Solanum tuberosum*) | StTFL1 | ABC24691.1 |
|  |  | StSP3D | BAV67096.1 |
|  |  | StSP6A | BAV67095.1 |
| 6 | Clustered gentian (*Gentia natriflora*) | GtTFL1 | BAK40196.1 |
| 7 | Chrysanthemum (*Chrysanthemum spp.*) | CsFTL3 | ACX48949.1 |
| 8 | Pumpkin (*Cucurbita maxima*) | Cm-FTL1 | ABI94605.1 |
|  |  | Cm-FTL2 | ABI94606.1 |
| 9 | Lombardy poplar (*Populus nigra*) | PnTFL1 | BAG12897.1 |
|  |  | PnFT1 | BAG12899.1 |
|  |  | PnFT2 | ABW73563.1 |
| 10 | Purple false brome (*Brachypodium distachyon*) | FTL1 | AHB63017.1 |
|  |  | FTL2 | XP_003564300.1 |
| 11 | Soybean  (Glycine max) | Dt1 | ADF31195.1 |
|  |  | GmMFT | NP_001236894 |
|  |  | GmFT2a | NP_001240185.1 |
|  |  | GmFT4 | NP_001276224 |
|  |  | GmFT5a | NP_001239994.1 |
| 12 | Tomato (*Solanum lycopersicum*) | SP | NP_001233974.2 |
|  |  | SFT | NP_001308081.1 |
| 13 | Rice (*Oryza sativa*) | RFT1 | NP_001408117.1 |
|  |  | Hd3a | NP_001408118.1 |
| 14 | Sugarcane (*Saccharum spp.*) | ScTFL1 | AEO72025.1 |
|  |  | ScFT1 | AHZ46121.1 |
| 15 | Snapdragon (*Antirrhinum spp.*) | CEN | Q41261.1 |
| 16 | Dancing lady orchid (*Oncidium gowerramsey*) | OnTFL1 | AIU44253.1 |
|  |  | OnFT | AIG63003.1 |
| 17 | Fig (*Ficus carica*) | FcFT1 | BAL22281.1 |
| 18 | Grapevine (*Vitis vinifera*) | VvTFL1A | ACY80737.1 |
|  |  | VvFT | ABF56526.1 |
| 19 | Japanese apricot (*Prunus mume*) | PmTFL1 | AEO72021.1 |
|  |  | PmFT | BAH82787.1 |
| 20 | Onion (*Allium cepa*) | AcFT1 | AGZ20207.1 |
|  |  | AcFT2 | AGZ20208.1 |
| 21 |  | AcFT4 | AGZ20210.1 |
| 22 | Kiwifruit (*Actinidia spp*.) | Kiwifruit CEN | AGK89940.1 |
| 23 | Lettuce (*Lactuca sativa*) | LsFT | BAK14369.1 |
| 24 | Sugar beet (*Beta vulgaris*) | BvFT1 | ADM92608.1 |
|  |  | BvFT2 | ADM92610.1 |
| 25 | London plane (*Platanus acerifolia*) | PaFT | AIG92770.1 |
| 26 | Black cherry (*Prunus serotina*) | PsTFL1 | AEO72024.1 |
| 27 | *Lotus japonicus* | Ljcen1 | AAQ93599.1 |
|  |  | LjFT | AFK38383.1 |
| 28 | Maize (*Zea mays*) | ZCN8 | AYA59799.1 |
|  |  | ZCN2 | ABX11004.1 |
| 29 | Medicago (*Medicago truncatula*) | MTFTa1 | AEI99551.1 |
|  |  | MtFTb1 | AEI99553.1 |
|  |  | MtFTc | AEI99555 |
| 30 | Orange (*Citrus sinensis*) | CsTFL | BAN13572.1 |
| 31 | Pea  (Pisum sativum) | PsFTa1 | ADZ05704.1 |
|  |  | PsFTa2 | ADZ05700.1 |
|  |  | PsFTb1 | ADZ05701.1 |
|  |  | PsFTb2 | ADZ05705.1 |
|  |  | PsFTc | ADZ05706.1 |
|  |  | PsTFL1a | AAR03725.1 |
|  |  | PsTFL1c | AAQ20811.1 |
| 32 | Peach (*Prunus persica*) | PpTFL1 | BAI99732.4 |
| 33 | Pear  (*Pyrus spp.*) | PpTFL1-2 | BAD10968.1 |
|  |  | PcTFL1-1 | BAD10963.1 |
|  |  | PcTFL1-2 | BAD10969.1 |
| 34 | Phalaenopsis orchid (*Phalaenopsis hybrid*) | PhFT | AFS60093.1 |
| 35 | Pharbitis (*Ipomoea nil*) | PnFT1a | BAG12899.1 |
|  |  | PnFT2a | BAD01561.1 |
| 36 | Physic nut (*Jatropha curcas*) | JcFT | AHX74040.1 |
| 37 | Poplar  (*Populus spp.*) | PtFT1 | AFU08239.1 |
|  |  | PtFT2 | AFU08240.1 |
|  |  | PopMFT | XP_002321507.1 |
| 38 | Ryegrass (*Lolium perenne*) | LpTFL1 | AAG31808.1 |
| 39 | Strawberry (*Fragaria vesca*) | FvFT1 | AEP23098.1 |
|  |  |  |  |
| 40 | Rose (Rosa spp.) | RoKSN | ADO64261.1 |
| 41 | Satsuma mandarin (*Citrus unshiu*) | CiFT | BAA77836.1 |
| 42 | Sunflower (*Helianthus annuus*) | HaFT1 | QIN85644.1 |
|  |  | HaFT4 | ADO61927.1 |
| 43 | Tobacco (*Nicotiana tabacum*) | NtFT1 | AFS17369.1 |
|  |  | NtFT2 | AFS17370.1 |
|  |  | NtFT3 | AFS17371.1 |
|  |  | NtFT4 | AFS17371.1 |
| 44 | Spring orchid (*Cymbidium spp.*) | CgFT | ADI58462.1 |
| 45 | Wheat (*Triticum aestivum*) | TaMFT | BAK78909.1 |
|  |  | TaFT | BBD19637.1 |
| 46 | Arabidopsis  (*Arabidopsis thaliana*) | AtATC | NP_180324.1 |
|  |  | AtTFL1 | AT5G03840.1 |
|  |  | AtBFT | XP_002864769.1 |
|  |  | AtFT | AT1G65480.1 |
|  |  | AtTSF | NP_193770.1 |
|  |  | AtMFT | NP_173250.1 |

**Table S3.** ***Cis*-regulatory elements analysis of 2.5-kb upstream promoter region of *DlFT1*, *LcFT1* and *NlFT1* predicted by MAST and** **PlantCARE.**

| **No.** | **Name of the elements** | **Core Sequences** | **Functional description ^(Xu et al. 2011; Yin et al. 2016)^** | ***DlFT1_pro_^In-321^*** | ***DlFT1_pro_^Del-321^*** | ***LcFT1_pro_*** | ***NlFT1_pro_*** |
| --- | --- | --- | --- | --- | --- | --- | --- |
| 1 | AAGAA-motif | GAAAGAA | Unknown | 0 | 1 | 0 | 0 |
| 2 | ABRE | ACGTG | *Cis*-acting element involved in theabscisic acid responsiveness. | 1 | 0 | 1 | 0 |
| 3 | ACE | CTAACGTATT | *Cis*-acting element involved in light responsiveness | 0 | 1 | 1 | 0 |
| 4 | AP-1 | TGAGTTAG | Stress response element *cis*-acting regulatory element | 0 | 0 | 0 | 2 |
| 5 | AT1-motif | AATTATTTTTTATT | part of a light responsive module | 0 | 0 | 2 | 0 |
| 6 | AT-rich element | ATAGAAATCAA | binding site of AT-rich DNA binding protein (ATBP-1) | 0 | 0 | 1 | 0 |
| 7 | Box 4 | ATTAAT | Part of a conserved DNA module involved in light responsiveness | 1 | 2 | 8 | 6 |
| 8 | Box III | atCATTTTCACt | protein binding site | 0 | 0 | 1 | 0 |
| 9 | CAAT-box | CAAT | Common *cis*-acting element in promoter and enhancer regions | 12 | 52 | 59 | 59 |
| 10 | CArG-box | CC[A/T]6GG | Binding motifs for MADS domain proteins  involved in flower formation | 1 | 0 | 0 | 0 |
| 11 | CArG-box | CC[A/T]7G/C[A/T]7GG | Binding motifs for MADS domain proteins  involved in flower formation | 0 | 1 | 2 | 2 |
| 12 | CGTCA-motif | CGTCA | *Cis*-acting regulatory element involved in the MeJA-responsiveness | 0 | 0 | 1 | 2 |
| 13 | chs-CMA2a | TCACTTGA | Part of a light responsive element | 0 | 1 | 0 | 1 |
| 14 | circadian | CAAAGATATC | *Cis*-acting regulatory element involved in circadian control | 0 | 0 | 1 | 0 |
| 15 | ERE | ATTTTAAA | Ethylene-responsive element | 1 | 1 | 1 | 1 |
| 16 | Gap-box | CAAATGAA(A/G)A | Part of a light responsive element | 0 | 0 | 0 | 1 |
| 17 | GATA-motif | AAGATAAGATT | Conserved in light-regulated and  tissue-specific expression genes | 0 | 0 | 1 | 0 |
| 18 | GATT-motif | CTCCTGATTGGA | part of a light responsive element | 0 | 1 | 1 | 0 |
| 19 | G-Box | CACGTT | *Cis*-acting regulatory elementinvolved in light responsiveness. | 1 | 0 | 1 | 0 |
| 20 | G-box | CACGTC | ubiquitous, *cis*-acting DNA  regulatory element | 0 | 0 | 0 | 1 |
| 21 | G-box | TAACACGTAG | ubiquitous, *cis*-acting DNA  regulatory element | 0 | 1 | 0 | 1 |
| 22 | GCN4_motif | TGAGTCA | Controlling seed-specific expression of the genes | 1 | 0 | 0 | 0 |
| 23 | GT1-motif | GGTTAA | light responsive element | 0 | 1 | 2 | 1 |
| 24 | HD-Zip 1 | CAAT(A/T)ATTG | element involved in differentiation of the palisade mesophyll cells | 0 | 0 | 1 | 0 |
| 25 | I-box | TGATAATGT | Part of a light responsive element | 0 | 1 | 0 | 0 |
| 26 | LAMP-element | CTTTATCA | part of a light responsive element | 0 | 0 | 0 | 1 |
| 27 | LTR | CCGAAA | *cis*-acting element involved in low-temperature responsiveness | 0 | 0 | 2 | 0 |
| 28 | MBSI | aaaAaaC(G/C)GTTA | MYB binding site involved in flavonoid biosynthetic genes regulation | 0 | 0 | 0 | 1 |
| 29 | MYB | CAACAG | Function as transcriptional activators in ABA-inducible gene  expression under drought stress | 0 | 2 | 2 | 0 |
| 30 | MYB | CAACTG | *Cis*-acting element involved in drought responsiveness | 0 | 2 | 6 | 2 |
| 31 | MYB | TAACTG | Function as transcriptional activators in ABA-inducible gene  expression under drought stress | 0 | 0 | 0 | 1 |
| 32 | MYB-like sequence | TAACCA | Function as transcriptional activators in ABA-inducible gene expression under drought stress | 0 | 0 | 2 | 0 |
| 33 | MYC | CAATTG | Drought and ABAresponses | 2 | 0 | 0 | 1 |
| 34 | MYC | TCTCTTA | *Cis*-acting element involved in drought responsiveness | 0 | 1 | 1 | 1 |
| 35 | MYC | CATTTG | Drought and ABA responses | 0 | 2 | 0 | 4 |
| 36 | MYC | CATGTG | *Cis*-acting element involved in drought responsiveness | 0 | 0 | 1 | 2 |
| 37 | TATA-box | TATATA | Core promoter element around 30 of transcription start | 21 | 71 | 130 | 62 |
| 38 | TCA | TCATCTTCAT | Salicylic acid responsive element | 0 | 1 | 1 | 0 |
| 39 | TCA-element | TCAGAAGAGG | Salicylic acid responsiveness | 0 | 1 | 1 | 0 |
| 40 | TCCC-motif | TCTCCCT | Part of a light responsive element | 0 | 0 | 1 | 0 |
| 41 | TCT-motif | TCTTAC | Part of a light responsive element | 0 | 0 | 1 | 1 |
| 42 | TGACG-motif | TGACG | *Cis*-acting regulatory element involved in the MeJA-responsiveness | 0 | 0 | 2 | 4 |
| 43 | W box | TTGACC | Binding site for WRKY transcription factor | 0 | 1 | 0 | 2 |
| 44 | WRE3 | CCACCT | A wound-responsive element | 0 | 2 | 2 | 1 |
| 45 | WUN-motif | TAATTACTC | A wound-responsive element | 0 | 0 | 1 | 0 |
| 46 | WUN-motif | TTATTACAT | Wound-responsive element | 0 | 0 | 0 | 1 |

**Table S4. *Cis*-** **regulatory elements analysis of 2.5-kb upstream promoter region of four *NlFT1s* predicted by MAST and PlantCARE.**

| **No.** | **Name of the elements** | **Core Sequences** | **Functional description ^(Xu et al. 2011; Yin et al. 2016)^** | ***NlFT1-a_pro_*** | ***NlFT1-b_pro_*** | ***NlFT1-c_pro_*** | ***NlFT1-d_pro_*** |
| --- | --- | --- | --- | --- | --- | --- | --- |
| 1 | AP-1 | TGAGTTAG | Stress response element | 2 | 1 | 1 | 1 |
| 2 | TATA-box | TATATAAA | Core promoter element around 30 of transcription start | 133 | 106 | 126 | 141 |
| 3 | Box 4 | ATTAAT | Part of a conserved DNA module involved in light responsiveness | 6 | 5 | 6 | 7 |
| 4 | CAAT-box | CAAT | Common *cis*-acting element in promoter and enhancer regions | 61 | 48 | 51 | 51 |
| 5 | CGTCA-motif | CGTCA | *Cis*-acting regulatory element involved in the MeJA-responsiveness | 2 | 1 | 2 | 1 |
| 6 | chs-CMA2a | TCACTTGA | Part of a light responsive element | 1 | 1 | 1 | 1 |
| 7 | ERE | ATTTTAAA | Ethylene-responsive element | 1 | 1 | 2 | 1 |
| 8 | Gap-box | CAAATGAA(A/G)A | Part of a light responsive element | 1 | 1 | 1 | 1 |
| 9 | GT1-motif | GGTTAAT | Light responsive element | 1 | 2 | 2 | 2 |
| 10 | LAMP-element | CTTTATCA | Part of a light responsive element | 1 | 1 | 1 | 1 |
| 11 | MBSI | aaaAaaC(G/C)GTTA | MYB binding site | 1 | 1 | 1 | 1 |
| 12 | MYBR | CAACCA | Function as transcriptional activators in ABA-inducible gene  expression under drought stress | 2 | 2 | 2 | 2 |
| 13 | MYBR | TAACTG | Function as transcriptional activators in ABA-inducible gene  expression under drought stress | 1 | 3 | 1 | 2 |
| 14 | MYC | CATTTG | Drought and ABA responses | 1 | 1 | 1 | 1 |
| 15 | MYC | TCTCTTA | *Cis*-acting element involved in drought responsiveness | 1 | 1 | 1 | 1 |
| 16 | MYC | CATGTG | *Cis*-acting element involved in drought responsiveness | 2 | 1 | 1 | 1 |
| 17 | MYC | CAATTG | *Cis*-acting element involved in drought responsiveness | 1 | 1 | 1 | 1 |
| 18 | TCT-motif | TCTTAC | Part of a light responsive element | 1 | 1 | 1 | 1 |
| 19 | TGACG-motif | TGACG | *Cis*-acting regulatory element involved in the MeJA-responsiveness | 4 | 2 | 4 | 2 |
| 20 | W box | TTGACC | Binding site for WRKY transcription factor | 1 | 1 | 1 | 1 |
| 21 | WRE3 | CCACCT | Unknown | 1 | 1 | 1 | 1 |
| 23 | CArG-box | CC[A/T]7G/C[A/T]7GG | Binding motifs for MADS domain proteins  involved in flower formation | 2 | 1 | 1 | 1 |
| 24 | WUN-motif | TTATTACAT | Wound-responsive element | 1 | 1 | 1 | 1 |
| 25 | WUN-motif | TAATTACTC | Wound-responsive element | 0 | 1 | 1 | 1 |
| 26 | MYBR | CAACAG | Function as transcriptional activators in ABA-inducible gene  expression under drought stress | 0 | 1 | 1 | 1 |
| 27 | MYBR | TAACCA | Function as transcriptional activators in ABA-inducible gene  expression under drought stress | 0 | 1 | 1 | 1 |
| 28 | ARE | AAACCA | *Cis*-acting regulatory element essential for the anaerobic induction | 0 | 1 | 1 | 1 |

**Table S5. Resequenced lychee accessions ^(^**^Hu et al. 2022^**^).^**

| **Accession name** | **Acc. ID** | **Status** | **Geographic origin** | **Fruit maturation period** |
| --- | --- | --- | --- | --- |
| YNW01 | YNW01 | Wild | Pingbian | Extremely early maturing |
| YNW02 | YNW02 | Wild | Pingbian | Extremely early maturing |
| YNW03 | YNW03 | Wild | Pingbian | Extremely early maturing |
| YNW04 | YNW04 | Wild | Jianshui | Extremely early maturing |
| YNW05 | YNW05 | Wild | Yuanyang | Extremely early maturing |
| YNW06 | YNW06 | Wild | Yuanyang | Extremely early maturing |
| YNW07 | YNW07 | Wild | Yuanyang | Extremely early maturing |
| YNW08 | YNW08 | Wild | Yuanyang | Extremely early maturing |
| VNW01 | VNW01 | Wild | Vietnam | Extremely early maturing |
| VNW02 | VNW02 | Wild | Vietnam | Extremely early maturing |
| VNW03 | VNW03 | Wild | Vietnam | Extremely early maturing |
| VNW04 | VNW04 | Wild | Vietnam | Extremely early maturing |
| GXDXW01 | GXDXW01 | Wild | Daxin | Extremely early maturing |
| GXDXW02 | GXDXW02 | Wild | Daxin | Extremely early maturing |
| Sanyuehong | SANYH | Cultivated | Guangdong | Extremely early maturing |
| Wengzao | WZ | Cultivated | Guangdong | Extremely early maturing |
| Guizao | GZ | Cultivated | Guangxi | Extremely early maturing |
| Hemaoli | HML | Cultivated | Yunnan | Extremely early maturing |
| Shuidong | SD | Cultivated | Guangdong | Early maturing |
| Siyuehong | SIYH | Cultivated | Guangdong | Early maturing |
| Zhongshanzhuangyuanhong | ZSZYH | Cultivated | Guangdong | Early maturing |
| Feizixiao | FZX | Cultivated | Guangdong | Early maturing |
| Caikengrouwa | CKRW | Cultivated | Fujian | Early maturing |
| Guiwei | GW | Cultivated | Guangdong | Late-maturing |
| Hehuadahongli | HHDHL | Cultivated | Guangdong | Late-maturing |
| Hexiachuan | HXC | Cultivated | Guangdong | Late-maturing |
| Huaizhi | HZ | Cultivated | Guangdong | Late-maturing |
| Liuyuexue | LYX | Cultivated | Guangdong | Late-maturing |
| Maguili | MGL | Cultivated | Guangdong | Late-maturing |
| MS48 | MS48 | Cultivated | Guangdong | Late-maturing |
| Nuomici | NMC | Cultivated | Guangdong | Late-maturing |
| Lingshanxiangli | LSXL | Cultivated | Guangxi | Late-maturing |
| Siliangguo | SLG | Cultivated | Guangxi | Late-maturing |
| Niuxinli | NXL | Cultivated | Hainan | Late-maturing |
| Wuheli | WHL | Cultivated | Hainan | Late-maturing |
| Yuanhe | YH | Cultivated | Hainan | Late-maturing |
| Zili | ZL | Cultivated | Hainan | Late-maturing |
| Ziniangxi | ZNX | Cultivated | Hainan | Late-maturing |
| Edanli | EDL | Cultivated | Hainan | Late-maturing |
| HNW01 | HNW01 | Wild | Wenchang | Late-maturing |
| HNW02 | HNW02 | Wild | Anding | Late-maturing |
| HNW03 | HNW03 | Wild | Hainan | Late-maturing |
| HNW04 | HNW04 | Wild | Wanning | Late-maturing |
| HNW06 | HNW06 | Wild | Sanya | Late-maturing |
| GXBBW01 | GXBBW01 | Wild | Bobai | Late-maturing |
| GXBBW02 | GXBBW02 | Wild | Bobai | Late-maturing |
| GXBBW03 | GXBBW03 | Wild | Bobai | Late-maturing |

**Table S6. Detailed information on the functional validation of *SVP* genes in 10 plant species.**

| **Species** | **Homologs** | **NCBI accession** |
| --- | --- | --- |
| Pepper （*Capsicum annuum*） | CaJOINTLESS | JQ698661 |
| Sweet cherries （*Prunus avium*） | PavSVP | XM_021955223 |
| Mango （*Mangifera indica*） | MiSVP3 | OP561443 |
|  | MiSVP4 | OP561444 |
|  | MiSVP1 | MZ542518 |
|  | MiSVP2 | MZ542519 |
| Potato (*Solanum tuberosum*) | STMADS16 | NP_001275284.1 |
| Brassica plants ( *Brassica rapa*) | BcSVP | Q06AL8 |
| Barley (*Hordeum vulgare*) | BM10 | EF043040.1 |
|  | BM1 | AJ249141.1 |
| Rice (*Oryza sativa*) | OsMADS22 | NP_001388916.1 |
|  | OsMADS47 | NP_001388995.1 |
| Eucalyptus (*Paulownia kawakamii*) | PkMADS1 | AF060880 |
| *Eucalyptus grandis* | EgrSVP | AY263809 |
| Arabidopsis *(Arabidopsis thaliana)* | AtAGL24 | OAO97218.1 |
|  | AtSVP | OAP09056.1 |

**Table S7. *SVP* genes identified in different species genome.**

| **Species** | **Gene ID** | **Name/clade** |
| --- | --- | --- |
| Lychee  (*Litchi chinensis*) | LITCHI001246 | LcSVP1 |
|  | LITCHI001247 | LcSVP2 |
|  | LITCHI001248 | LcSVP3 |
|  | LITCHI001249 | LcSVP4 |
|  | LITCHI001250 | LcSVP5 |
|  | LITCHI001251 | LcSVP6 |
|  | LITCHI001252 | LcSVP7 |
|  | LITCHI001256 | LcSVP8 |
|  | LITCHI007230 | LcSVP9 |
|  | LITCHI010652 | LcSVP10 |
| Soybean  (*Glycine max*) | Glyma.02G041500.1 | SVP1 |
|  | Glyma.01G023500.1 | SVP1 |
|  | Glyma.13G257000.1 | SVP2 |
|  | Glyma.08G068200.8 | SVP3 |
|  | Glyma.15G058000.1 | SVP3 |
|  | Glyma.15G057900.2 | SVP3 |
|  | Glyma.07G181600.1 | SVP3 |
|  | Glyma.13G256900.1 | SVP3 |
|  | Glyma.06G095700.1 | SVP3 |
| Tomato （*Solanum lycopersicum* ） | Prupe.6G199000.1 | SVP3 |
|  | Solyc01g105800.4.1 | SVP3 |
| Mandarin (*Clementine mandarin*) | Ciclev10026457m | SVP1 |
|  | Ciclev10002707m | SVP2 |
|  | Ciclev10029491m | SVP3 |
| White poplar （*Populus tomentosa*） | Potri.007G010800.7 | SVP1 |
|  | Potri.002G105600.1 | SVP2 |
|  | Potri.005G155300.4 | SVP2 |
|  | Potri.007G115100.6 | SVP3 |
|  | Potri.017G044200.1 | SVP3 |
|  | Potri.007G115200.7 | SVP3 |
|  | Potri.017G044500.1 | SVP3 |
|  | Potri.007G115000.7 | SVP3 |
| Peach （ *Prunus persica*） | Solyc11g010570.2.1 | SVP1 |
|  | Prupe.1G531400.1 | SVP2 |
|  | Prupe.1G531600.1 | SVP2 |
|  | Prupe.1G531700.1 | SVP2 |
|  | Prupe.1G531100.2 | SVP2 |
|  | Prupe.8G069300.1 | SVP3 |
| Cabbage (*Brassica oleracea*) | Bol044741 | SVP1 |

**Table S8. qRT-PCR primer sequences.**

| **Abbreviation** | **Gene ID** | **Forward primers（5ˊ→3ˊ）** | **Reverse primers（5ˊ→3ˊ）** |
| --- | --- | --- | --- |
| *Dlactin* | Dil.11g013910.1 | TGCTATCCTTCGGTTGGACC | CGGACGATTTCCCGTTCAG |
| *DlFT2* | Dil.05g001570.1 | ATGAGAGCCCCAGACCAACTGT | ATTCACTGTCTGCCTGCCTT |
| *DlFT1* | Dil.05g001470.1 | TGAGCTCAAACCCTCTCAGA | ACCGGAGATCCAAGGTTGTA |
| *LcSVP9* | LITCHI007230 | GTTCTTTGCGATGCCGATGT | AACCTGGTGTAGTTGCTGTTCTC |
| *AtActin* | AT5G09810 | GCTGACCGTATGAGCAAAGA | GATCCTCC GATCC AGAC ACT |
| *AtFT* | AT1G65480 | AGGTGACTAATGGCTTGGATCTAA | GGAGATATTCTCGGAGGTGAGG |
